# Ferrous Sulfate Supplementation Causes Significant Gastrointestinal Side-Effects in Adults: A Systematic Review and Meta-Analysis

**DOI:** 10.1371/journal.pone.0117383

**Published:** 2015-02-20

**Authors:** Zoe Tolkien, Lynne Stecher, Adrian P. Mander, Dora I. A. Pereira, Jonathan J. Powell

**Affiliations:** 1 MRC Human Nutrition Research, Elsie Widdowson Laboratory, Cambridge, United Kingdom; 2 MRC Biostatistics Unit, Institute of Public Health, Robinson Way, Cambridge, United Kingdom; RWTH Aachen, GERMANY

## Abstract

**Background:**

The tolerability of oral iron supplementation for the treatment of iron deficiency anemia is disputed.

**Objective:**

Our aim was to quantify the odds of GI side-effects in adults related to current gold standard oral iron therapy, namely ferrous sulfate.

**Methods:**

Systematic review and meta-analysis of randomized controlled trials (RCTs) evaluating GI side-effects that included ferrous sulfate and a comparator that was either placebo or intravenous (IV) iron. Random effects meta-analysis modelling was undertaken and study heterogeneity was summarised using *I^2^* statistics.

**Results:**

Forty three trials comprising 6831 adult participants were included. Twenty trials (n = 3168) had a placebo arm and twenty three trials (n = 3663) had an active comparator arm of IV iron. Ferrous sulfate supplementation significantly increased risk of GI side-effects versus placebo with an odds ratio (OR) of 2.32 [95% CI 1.74–3.08, *p*<0.0001, *I^2^* = 53.6%] and versus IV iron with an OR of 3.05 [95% CI 2.07-4.48, *p*<0.0001, *I^2^* = 41.6%]. Subgroup analysis in IBD patients showed a similar effect versus IV iron (OR = 3.14, 95% CI 1.34-7.36, *p* = 0.008, *I^2^* = 0%). Likewise, subgroup analysis of pooled data from 7 RCTs in pregnant women (n = 1028) showed a statistically significant increased risk of GI side-effects for ferrous sulfate although there was marked heterogeneity in the data (OR = 3.33, 95% CI 1.19-9.28, *p* = 0.02, *I^2^* = 66.1%). Meta-regression did not provide significant evidence of an association between the study OR and the iron dose.

**Conclusions:**

Our meta-analysis confirms that ferrous sulfate is associated with a significant increase in gastrointestinal-specific side-effects but does not find a relationship with dose.

## Introduction

In the UK, iron deficiency anemia (IDA) affects around 4.7 million people every year [1,2]. Groups mostly at risk include those with increased iron demands, for example children and pregnant women [3,4,5], and especially those with increased iron loses, for example pre-menopausal women [6] and patients with inflammatory bowel disease (IBD) [7,8].

First-line treatment is oral therapy with ferrous iron (Fe(II)) salts [9]. For example, in 2012 more than 6.8 million prescriptions were filled for oral iron in England and 97.6% of them were for simple Fe(II) salts [10]. Gastrointestinal side-effects are the most commonly reported adverse effects associated with oral iron treatment and include nausea, flatulence, abdominal pain, diarrhoea, constipation, and black or tarry stools [11,12,13,14,15]. For very many years patient side-effects have been the main concern with oral iron therapy [16] but, recently, studies have consistently shown that soluble oral iron also negatively impacts the colonic microbiota, promoting the presence of potentially pathogenic bacteria at the expense of beneficial bacteria [17,18,19]. Most recently there have been concerns over ‘available’ iron in the colon as a risk factor for inflammatory signalling and colorectal carcinogenesis [18,20]. Nonetheless, irrespective of the mechanistic foundation of the GI side-effects that appear to be related to oral iron therapy, the impact results in non-adherence in up to 50% of patients. This leads to significant treatment failures and unnecessary follow-up investigations [11,21,22,23,24,25,26,27,28,29,30].

In trying to capture the true incidence of GI side-effects with oral iron therapy, discrepancies between studies and limitations of study design have made overall conclusions difficult to reach [11,14,15]. A recent review has tried to capture data for adverse events from studies with oral iron supplementation in patients without gastrointestinal disease and reported overall adverse event incidences of 32.3% for ferrous sulfate, 47% for ferrous fumarate and 30.9% for ferrous gluconate [11]. However, the meta-analysis reported herein has refined the study eligibility criteria in relation to the prior review [11]. Notably, we have only included randomized controlled studies with a ferrous sulfate intervention arm; we restricted adverse events to the common focus of concern with therapeutic oral iron, namely GI side-effects, and we have only included studies with a common comparator arm, which was either placebo or intravenous iron.

Since ferrous sulfate is the gold standard (most commonly prescribed) oral iron therapy in the UK and many other countries [10,31], our aim was to quantify the odds ratio for oral ferrous sulfate- associated gastrointestinal side-effects versus placebo or IV iron. To this end, we have carried out a systematic review and meta-analysis of all published randomized controlled trials (RCTs) reporting gastrointestinal-specific side-effects that have included ferrous sulfate against placebo or IV iron. We have performed sub-group analysis for pregnant women and patients with IBD since these are two population groups at higher risk of iron deficiency anemia and for whom sufficient robust data were likely to be available [14,32,33]. Finally, we explored whether the iron dose is associated with the odds of gastrointestinal side-effects using meta-regression analysis.

## Methods

### Search strategy

The following bibliographic online databases were searched (last search in March 2014) for all dates up to and including December 2013: MEDLINE via PubMed (http://www.ncbi.nlm.nih.gov/pubmed), Cochrane Library (http://www.thecochranelibrary.com/), EMBASE (http://www.elsevier.com/online-tools/embase), ISI web of Science (http://wok.mimas.ac.uk/), SCOPUS (http://www.scopus.com/), Current Controlled Trials (CCT) (http://www.controlled-trials.com/), International Standard Randomized Controlled Trial Number (ISRCTN) Register (http://www.controlled-trials.com/isrctn/), WHO International Clinical Trials Registry Platform (ICTRP) (http://www.who.int/ictrp/en/), ProQuest Dissertations and Thesis (http://www.proquest.co.uk/en-UK/catalogs/databases/), ClinicalTrials.gov (http://clinicaltrials.gov/).

Publications reporting randomized controlled studies with ferrous sulfate were identified by using the search terms *ferrous*, *ferrous sulphate*, *ferrous sulfate*, *iron supplement*, *ferrous salt*, *iron*, paired with the terms *randomized controlled trial*, *randomized controlled trial*, *controlled trial*, *controlled clinical study*. No limits on language or publication type were imposed. Please refer to S1 File in the online issue for the full search strategy.

Additionally, the following Institutions were contacted (in January 2012) via email to request any additional studies not identified through the electronic search: WHO department of Nutrition for Health and Development (NHD) as well as 12 WHO regional offices (Africa, Americas, South East Asia, Europe, Eastern Mediterranean, Western Pacific, International Agency Research on Cancer (IARC), Centre for Health Development, Lyon office, Mediterranean Centre for Health Risk Reduction, Office at United Nations), Center for Disease Control and Prevention, United Nations Children’s Fund (UNICEF), World Food Programme (WFP), the Micronutrient Initiative, and Sight and Life Foundation. No protocol exists for this systematic review.

### Eligibility criteria

Studies were selected for analysis if they met the following criteria: (1) the study was an RCT conducted in adult human participants with either a parallel or crossover design, (2) the study included an arm/group with ferrous sulfate, (3) the study included a comparator arm/group with either placebo or intravenous iron and (4) gastrointestinal side-effects were recorded separately for each arm (i.e. ferrous sulfate and comparator). Where co-interventions were administered with the ferrous sulfate arm (for example ascorbic acid or folic acid) the studies were only included if the same co-intervention was administered in the comparator arm and when in the authors’ view the co-interventions should not influence side-effects in either arm.

### Selection and data extraction

Titles of all records retrieved were initially screened by ZT. All potentially relevant studies were then further screened as abstracts by three independent assessors. Three authors (ZT, LS and DP) independently screened full texts of all potentially relevant papers to ensure they met the inclusion criteria.

Data were extracted independently by two authors (ZT and LS) and discrepancies were resolved before data were compiled into a database that included the following fields: (1) patient population, (2) inclusion age criteria, (3) mean age, (4) participant numbers, (5) commercial name for ferrous sulfate (if any), (6) ferrous sulfate dose, (7) frequency of dose, (8) equivalent iron content, (9) duration of the intervention, (10) hemoglobin changes from baseline to end of the intervention, (11) gastrointestinal side-effects reported, (12) comparator group (placebo or intravenous iron), (13) co-interventions. In most studies, patient numbers used for the meta-analysis were those that were randomized, with the exception of studies that specified a subset safety population (i.e. if it was stated that there was only a subset of patients where adverse effects were assessed). If side-effects were reported multiple times during the trial, then only measures at the end of the intervention period were included in the meta-analysis. Data were extracted at the participant level, i.e the number and/or percentage of participants with at least one GI side-effect within a study arm. The ‘total number of participants’ was taken to be the number within the safety (i.e. side-effect assessment) population when specified, otherwise it was taken as the number of participants randomized. Some studies reported the number of participants experiencing each type of GI side-effect and not overall. In these cases, the commonest GI symptom was deemed (conservatively) to represent the number of participants that experienced GI side-effects. For example, in the study of Mirrezaie *et al* [34], 5 participants in the ferrous sulfate arm reported heartburn, 17 reported nausea, 2 reported abdominal cramps and 1 reported constipation, so the number of participants in the ferrous sulfate arm with a GI side-effect was taken to be 17.

The generated database for all of the above is available upon request.

### Statistical analysis

Studies that met the eligibility criteria were included in the meta-analysis. The random-effects model proposed by DerSimonian and Laird [35] was used for the meta-analysis. The odds in each arm were calculated as *p/(1-p)* where *p* is the proportion with GI side- effects. The odds ratio (OR) was calculated as the odds in the ferrous sulfate arm divided by the odds in the comparator arm. The meta-analyses used study specific log odds ratios as the outcome and the resulting pooled estimates were converted to OR. Values for the OR above 1 indicate that ferrous sulfate is associated with more side-effects in comparison to either placebo or intravenous iron. Risk of bias in individual studies was assessed independently by two co-authors based upon the Cochrane Collaboration’s Tool [36]. Furthermore, we assessed risk of bias connected to how the studies obtained information about gastrointestinal adverse-events (questionnaire, face to face interviews, spontaneous reporting by participants etc.). Publication bias was assessed by ‘funnel plots’ of the effect [log (OR)] against its standard error. The symmetry of the plots was assessed visually to look for asymmetry. Heterogeneity was assessed using the *I* ^*2*^ statistic and substantial heterogeneity exists when *I* ^*2*^>50% [37]. Subgroup analyses were carried out for two pre-defined subgroups, namely pregnant women and patients with inflammatory bowel disease. When zero GI side-effects were reported for the placebo or IV comparator arm a standard correction of adding 0.5 of a side-effect to each arm was used to enable calculation of the study-specific log OR. However, since this was a relatively common occurrence in the studies with an IV iron comparator, the meta-analysis was repeated excluding those studies where less than one gastrointestinal side-effect was reported in the IV iron arm.

We produced forest plots to visualise the ORs and 95% CIs of each study, each subgroup and all studies combined. For studies with a cross-over design both cross-over periods were treated independently in the analysis because there was insufficient information regarding individual tabulation of side-effects to apply the methodology proposed by Elbourne *et al*. [38].

Furthermore, meta-regression analyses were conducted to determine if the odds of gastrointestinal side-effects with ferrous sulfate was greater with higher doses. We produced bubble plots where individual studies are represented by circles, with the size of the circle being inversely proportional to the variance of the estimated odds ratio used in the meta-regression (i.e. the larger the circle, the more precise is the estimated OR). For the most commonly reported gastrointestinal side effects in the oral ferrous sulfate group, a random-effects meta-analysis (using log-transformed odds) was performed. This provided a pooled estimate of the incidence of each symptom in participants taking oral ferrous sulfate.

Meta-analyses were done with STATA 13 (StataCorp LP, Texas, USA).

## Results

### Study characteristics

**Fig 1:**
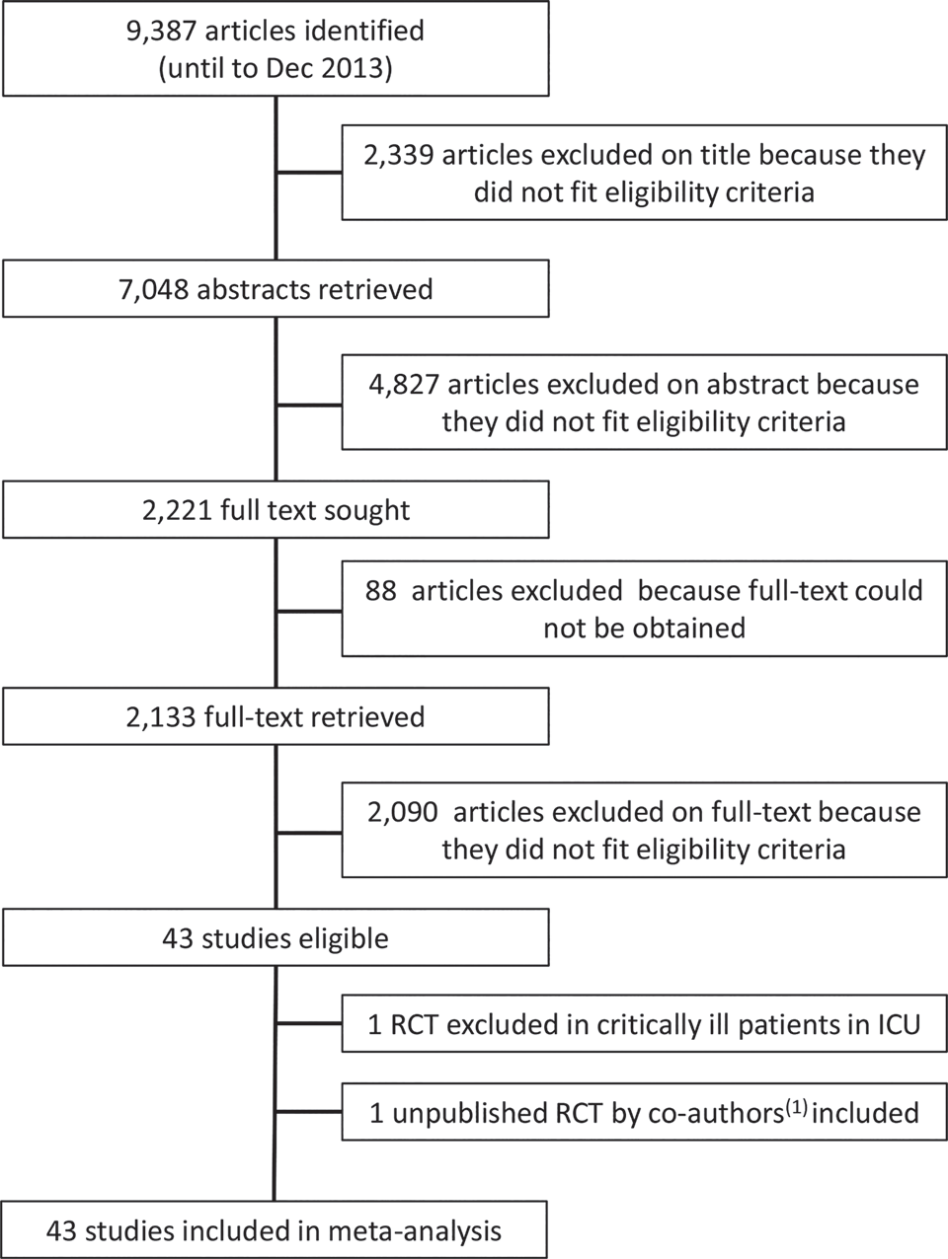
Study flow diagram. RCT, randomized controlled trial; ICU, intensive care unit. (1) This study was carried out by the co-authors and is currently submitted for publication and under review. A list compiling the 88 references that were not obtained is provided in Table A in S1 File.

A total of 44 studies with publication dates ranging from 1966 to 2013 met the inclusion criteria (Fig. 1). One study was excluded from the final meta-analysis because it was conducted in critically ill surgical patients in intensive care so ferrous sulfate was mixed in the enteral feed rather than being delivered in pure supplemental form as in the other studies [39]. Tables 1 and 2 summarise the characteristics of the 43 studies (n = 6831 participants overall) included in the meta-analyses. Twenty studies (n = 3168 participants) evaluated side-effects associated with oral ferrous sulfate (20–222 mg Fe/day) against a placebo comparator (Table 1) and 23 studies (n = 3663 participants) evaluated side-effects with oral ferrous sulfate (100–400 mg Fe/day) against an intravenous iron comparator (Table 2). We conducted 2 independent meta-analysis, one for the placebo-controlled trials and one for the IV iron-controlled trials, due to clear differences in trial design and study populations, namely: (i) the placebo-controlled trials were generally double-blind with healthy non-anaemic participants whilst (ii) the IV iron-controlled trials were open-label with moderate-severely anaemic patients. The result of the assessment of risk of bias for the individual studies is presented in Table A in S1 File. Included is also the assessment of variability in the methodology used to collate information for gastrointestinal-related adverse-effects.

**Table 1:** Randomized controlled trials with a placebo comparator arm/group included in the meta-analysis.

First author, year	Study	Participants(^2^)	Age	Duration	Iron dose^(^ ^3^ ^)^	FeSO_4_		placebo		Baseline Hb (g/dL)
(reference)	design^(^ ^1^ ^)^	(mean)	(weeks)	(mg/day)	n	GISEs n (%)	n	GISEs n (%)	(FeSO_4_)
**Baykan, 2006** [62]	Parallel	F	27.8	17.3	80	82	19 (23)	86	16 (19)	12.9
**Cook, 1990** [63]	Parallel	F	18–48	2	50	67	31 (46)	66	14 (21)	NR
**Davis, 2000** [64]	Parallel	RLS	58.6	12	130	14	5 (36)	14	0 (0)	14.3
**Fouad, 2013** [65]	Parallel	F	35	1	25	20	8 (40)	20	4 (20)	NR
**Ganzoni, 1974** [66]	Cross-over	M+F	27M, 33F	2	111	90	49 (54)	90	19 (21)	15.25
**Gordeuk, 1987** [67]	Parallel	F, blood donors	NR	1	180	24	18 (75)	23	8 (35)	12.7
**Hallberg, 1966**_1 [24]	Parallel	Blood donors	NR	NR	222	175	40 (23)	169	23 (14)	NR
**Hallberg, 1966**_2 [24]	Parallel	Blood donors	NR	NR	222	111	31 (28)	115	16 (14)	NR
**Hallberg, 1966**_3 [24]	Parallel	Blood donors	NR	NR	180	170	45 (26)	177	22 (12)	NR
**Levy, 1978** [68]	Cross-over	M+F	19–55	4.3	200	107	57 (53)	107	22 (21)	NR
**Maghsudlu, 2008** [69]	Parallel	F, blood donors	28.7	4	150	185	19 (10)	182	8 (4)	13.52
**Mirrezaie, 2008** [34]	Parallel	F, blood donors	34.2	8	50	49	17 (35)	46	9 (20)	NR
**Meier, 2003** [70]^**(4)**^	Parallel	Pregnancy	25.2	NR	60	38	24 (63)	36	19 (53)	13
**Makrides, 2003** [71]	Parallel	Pregnancy	28.5	20	20	200	136 (68)	193	133 (69)	13.1
**Pereira,** [40]	Parallel	M+F	32	1	130	10	9 (90)	10	4 (40)	NR
**Sutton, 2004** [41]	Parallel	Hip and knee-replacement	70	6	195	35	8 (23)	37	8 (22)	10.4
**Tuomainen, 1999** [72]^**(5)**^	Parallel	M	45–64	26	180	15	3 (20)	15	0 (0)	14.53
**Vaucher, 2012** [60]	Parallel	F	36.5	12	80 slow-Fe	102	12 (12)	96	10 (10)	13.5
**Yalcin, 2009** [73]	Parallel	Post-partum	27.7	15.3	80	24	8 (33)	23	6 (26)	13.1
**Waldvogel, 2012** [59]	Parallel	F, blood donors	31.8	4	80 slow-Fe	74	25 (34)	71	8 (11)	12.6

Two out of the 20 studies contained a co-intervention in both arms as indicated.

Abbreviations: M, male; F, female; NR, not reported or unclear; RLS, restless led syndrome; GISEs, gastrointestinal side-effects shown as percentage of patients that experience gastrointestinal side-effects; Hb, hemoglobin; FeSO_4_, ferrous sulfate group.

^(1)^ All trials were double-blind except Maghsudlu, 2008 [69]

^(2)^ All participants were generally healthy and non-anaemic with the exception of Sutton, 2004 [41]

^(3)^ Unless indicated all trials used standard ferrous sulfate (i.e. not modified-release) and daily posology. Tardyferon® used in studies [59,60]

^(4)^ Co-intervention: folic acid in both groups.

^(5)^ Co-intervention: ascorbic acid in both groups.

**Table 2:** Randomized controlled trials with an intravenous iron comparator arm/group included in the meta-analysis.

First author, year	Study	Participants	Age	Duration	Iron dose^(^ ^1^ ^)^	FeSO_4_		IV		Baseline Hb g/dL
(reference)	design		(mean)	(weeks)	(mg/day)	n	GISEs n (%)	n	GISEs n (%)	(FeSO_4_)^(^ ^2^ ^)^
**Agarwal, 2006** [74]	Parallel	Non-dialysis CKD	62.3	6	195	45	9 (20)	44	13 (30)	10.7
**Auerbach, 2004** [75]^(^ ^3^ ^)^	Parallel	Cancer patients	66	6	130	43	1 (2)	78	0 (0)	9.7
**Bhandal, 2006** [76]	Parallel	Post-partum	28	6	130	21	7 (33)	22	0 (0)	7.5
**Breymann, 2008** [77]	Parallel	Post-partum	27.5	12	200	117	12 (10)	227	8 (4)	9.76
**Charytan, 2005** [78]^(^ ^3^ ^)^	Parallel	CKD	60	4.1	195	48	17 (35)	48	6 (13)	9.7
**Guerra Merino, 2012** [61]	Parallel	Post-partum	30	6	120 slow-Fe	7	2 (29)	6	0 (0)	8.6
**Henry, 2007** [79]^(^ ^3^ ^)^	Parallel	Cancer	65.3	8	195	61	24 (39)	63	24 (38)	10.3
**Mudge 2012** [80]	Parallel	Kidney transplant	46.4	3	210	51	6 (12)	51	3 (6)	9.8
**Seid, 2008** [81]	Parallel	Post-partum	26.5	6	195	147	16 (11)	142	3 (2)	8.88
**Strickland, 1977** [58]	Cross-over	Dialysis CKD	NR	26	100 slow-Fe	20	2 (10)	20	0 (0)	8.03
**Tokars, 2010** [82]	Parallel	CKD	NR	8	195	91	11 (12)	91	7 (8)	≤ 11
**Van Wyck, 2005** [83]	Parallel	Non-dialysis CKD	63.9	8	195	91	16 (18)	91	8 (9)	10.1
**Van Wyck, 2007** [84]	Parallel	Post-partum	26.1	6	195	178	43 (24)	174	11 (6)	9
**Van Wyck, 2009** [85]	Parallel	Heavy menorrhagia	39.5	6	195	226	32 (14)	230	8 (3)	9.4
**Kochhar, 2013** [86]^(^ ^4^ ^)^	Parallel	Pregnancy (24–34 wk)	23	4	180	50	4 (8)	50	2 (4)	7.6
**Vazquez Pacheco, 1980** [87]^(^ ^5^ ^)^	Parallel	Pregnancy	26	4	195	20	4 (20)	20	0 (0)	7.76
**Al-Momen, 1996** [88]	Parallel	Pregnancy (<32 wk)	27.6	6.9	180	59	18 (31)	52	0 (0)	7.66
**Bayoumeu, 2002** [56]^**(6)**^	Parallel	Pregnancy (24 wk)	23	4	240 slow-Fe	25	1 (4)	25	0 (0)	9.7
**Bencaiova, 2009** [57]	Parallel	Pregnancy (15–20 wk)	Range 15–42	NR	80 slow-Fe	130	23 (18)	130	0 (0)	12.4
**Kulnigg, 2008** [89]	Parallel	IBD	47	12	200	63	4 (6)	137	4 (3)	9.1
**Lindgren, 2009** [30]	Parallel	IBD	42.8	20	400	46	11 (24)	45	0 (0)	10.38
**Reinisch, 2013** [90]	Parallel	IBD	Median 35	8	200	109	4 (4)	223	3 (1)	9.61
**Schroder, 2005** [91]	Parallel	IBD	Median 33	6	100	24	5 (21)	22	2 (9)	9.6

Six out of the 23 studies contained a co-intervention in both arms as indicated.

Abbreviations: M, male; F, female; CKD, chronic kidney disease; IBD, inflammatory bowel disease; NR, not reported or unclear; GISEs, gastrointestinal side effects shown as percentage of patients that experience gastrointestinal side-effects; Hb, hemoglobin;FeSO_4_, ferrous sulfate group; IV, intravenous iron group; slow-Fe, modified-release ferrous sulfate.

^(1)^ Iron dose in the FeSO4 group, unless indicated all trials used standard ferrous sulfate (i.e. not modified-release) and daily posology. Tardyferon® used in studies [56,57,61] and Ferrogradumet-Abbot used in study [58].

^(2)^ There was no statistically significant difference in baseline hemoglobin between the ferrous sulfate and the IV iron arms/groups.

^(3)^ Co-intervention: recombinant erythropoietin.

^(4)^ Co-intervention: mebendazole and folic acid.

^(5)^ Co-intervention: vitamin B12 and folic acid.

^(6)^ Co-intervention: folic acid.

Publication bias was investigated with ‘funnel plots’, see Figure A in S1 File. The outliers in the funnel plots corresponded to studies reporting zero GI side-effects either in the IV iron-arm (9 studies) or placebo-arm (2 studies) (refer to Tables 1 and 2) and to 1 small (n = 10) placebo-comparator study [40]. The pattern of the remaining studies, although not following the expected funnel shape, is symmetrical and therefore does not provide evidence of publication bias.

### Ferrous sulfate versus placebo

**Fig 2:**
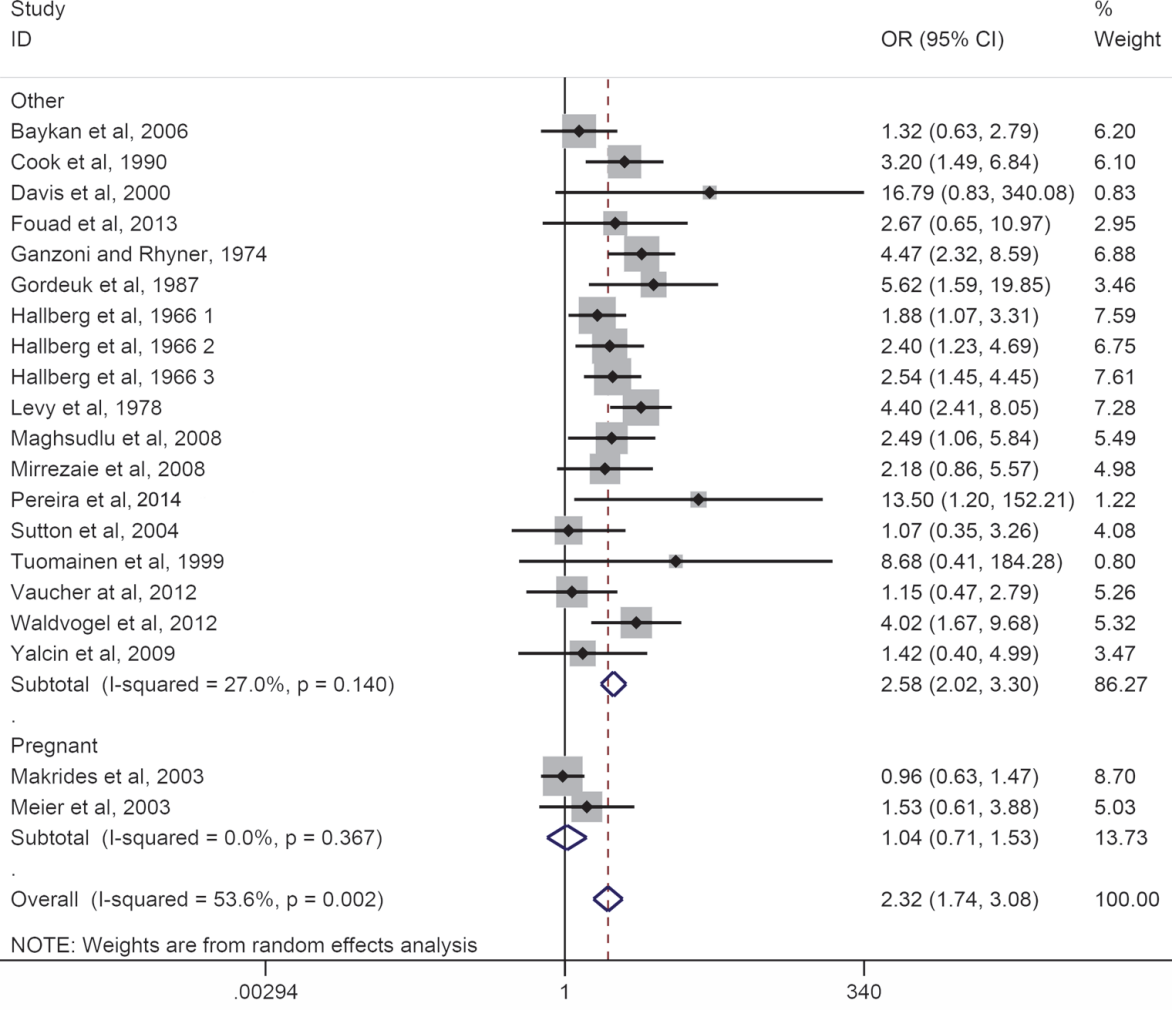
Forest plot for the effect of daily ferrous sulfate supplementation on the incidence of gastrointestinal side-effects in placebo-controlled RCTs. Data for random-effects meta-analysis are shown. For each study the closed diamond represents the mean estimated effect and the horizontal lines the 95% CI. The grey shaded area surrounding each closed diamond represents the weight of each study in the analysis. Weight was assigned based on the (inverse of) the sum of the within-study variance and between study variance. Open diamonds represent the subgroup mean difference and pooled overall mean differences as shown. Test for overall effect: z-score = 7.54 (other), 0.20 (pregnant), 5.79 (overall); *p*-value <0.0001 (other), = 0.8 (pregnant), <0.0001 (overall). OR, odds ratio; CI, confidence interval. Data shown for 20 RCTs (n = 3168).

For the 20 trials that included placebo as the comparator a significant increase in the incidence of gastrointestinal side-effects was observed with ferrous sulfate OR = 2.32 [95% CI 1.74–3.08, *p*<0.0001, *I* ^*2*^ = 53.6%] (Fig. 2). Nineteen of these 20 placebo-controlled trials were conducted in healthy non-anaemic individuals and therefore hemoglobin repletion was not a primary outcome. The only placebo-controlled trial in anaemic participants was carried out in patients who became anaemic following joint replacement surgery and reported an average increase in hemoglobin of 1.94 g/dl (range -0.3 to +4.2) following 6 weeks of ferrous sulfate therapy compared to an increase of 1.63 g/dl (range -1 to + 3.6) with placebo [41].

### Ferrous sulfate versus IV iron

**Fig 3:**
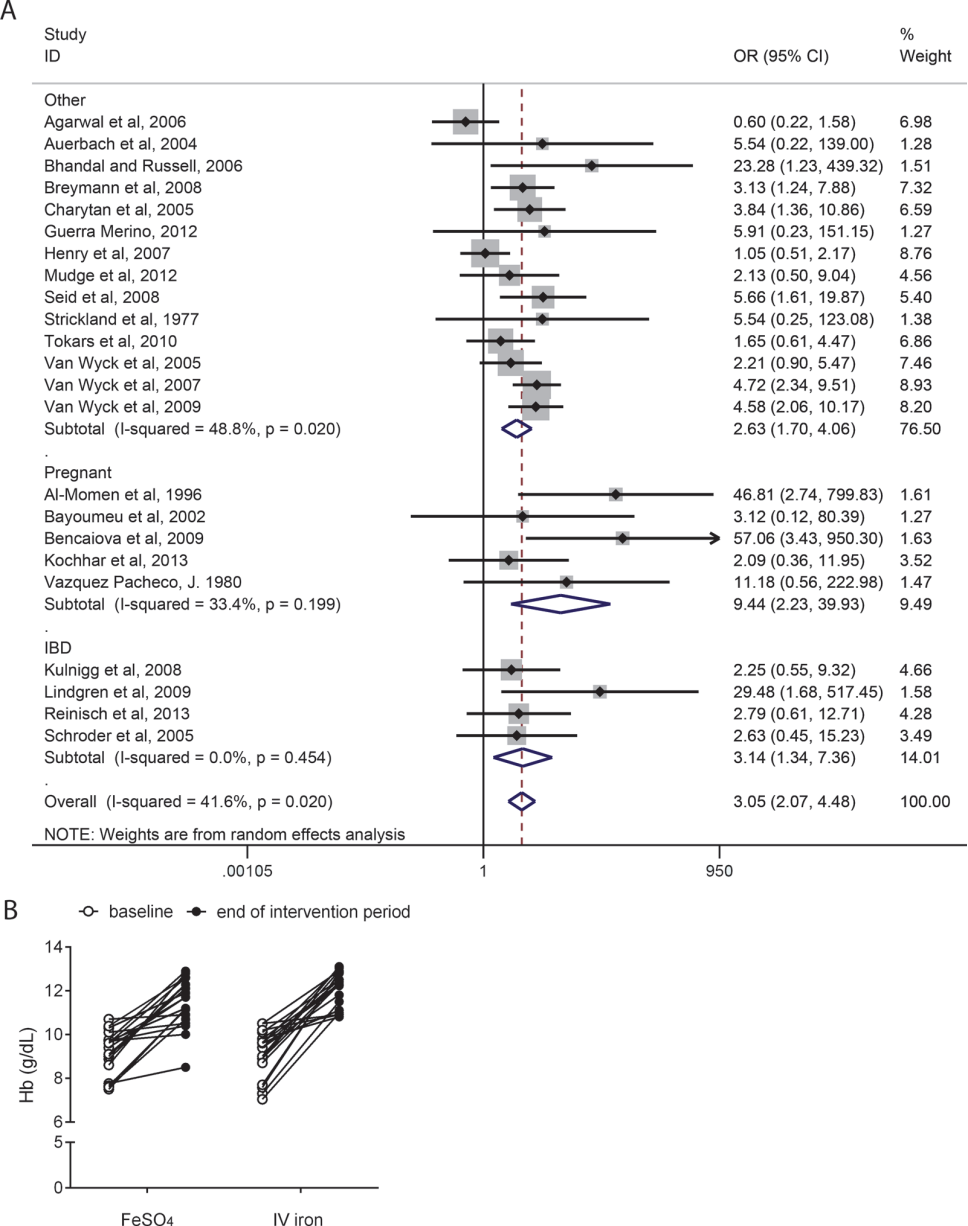
Effect of daily ferrous sulfate supplementation on the incidence of gastrointestinal side-effects and hemoglobin repletion in intravenous iron-controlled RCTs. **A,** Forest plot for random-effects meta-analysis of the effect of ferrous sulfate supplementation on the incidence of gastrointestinal side-effects against intravenous iron. For each study the closed diamond represents the mean estimated effect and the horizontal lines the 95% CI. The grey shaded area surrounding each closed diamond represents the weight of each study in the analysis. Weight was assigned based on (inverse of) the sum of the within-study variance and between study variance. Open diamonds represent the subgroup mean difference and pooled overall mean differences as shown. Test for overall effect: z-score = 4.36 (other), 3.05 (pregnant), 2.63 (IBD), 5.67(overall); *p*-value <0.0001 (other), = 0.002 (pregnant), = 0.008 (IBD), <0.0001 (overall). OR, odds ratio; CI, confidence interval. **B,** Hemoglobin increase in both ferrous sulfate (FeSO_4_) and intravenous iron (IV iron) arms from baseline (open circles) to end of study intervention (closed circles). Data shown for 20 RCTs (n = 3261).

For the 23 studies that included intravenous iron as the comparator a significant increase in the incidence of gastrointestinal side-effects was observed with ferrous sulfate with an OR = 3.05 [95% CI 2.07–4.48, *p*<0.0001, *I* ^*2*^ = 41.6%] (Fig. 3A). Furthermore, mean hemoglobin increase was reported for 20 of the 23 eligible IV iron-controlled trials (Fig. 3B and Table B in S1 File). Overall, for these 20 trials, the mean increase in hemoglobin for the ferrous sulfate arm was lower than for the IV iron arm although formal comparative analysis was not undertaken being outside the *a priori* objectives of our analysis (Fig. 3B).

The IBD subgroup analysis (4 studies, n = 669 participants) also showed a significantly higher incidence of gastrointestinal side-effects in the ferrous sulfate arm than in the IV iron arm with OR = 3.14 [95% CI 1.34–7.36, *p* = 0.008, *I* ^*2*^ = 0%] (Fig. 3A). The same was observed in the pregnancy subgroup analysis (5 studies, n = 561 participants), with OR = 9.44 [95% CI 2.23–39.93, *p* = 0.002, *I* ^*2*^ = 33.4%] (Fig. 3A).

Nine out of the 23 IV-iron comparator studies reported zero GI side-effects in the IV-iron arm so, as detailed in Methods, we added the standard correction of 0.5 of a side-effect to each arm to enable calculation of the study-specific log odds ratio. Nevertheless, when we excluded these 9 studies from the meta-analysis the same overall effect was observed with an OR = 2.41 (95% CI 1.66–3.50, *p*<0.0001, *I* ^*2*^ = 42.1%) for ferrous sulfate (*see*Figure B in S1 File).

### Ferrous sulfate in pregnancy: placebo- and IV iron-controlled trials combined

**Fig 4:**
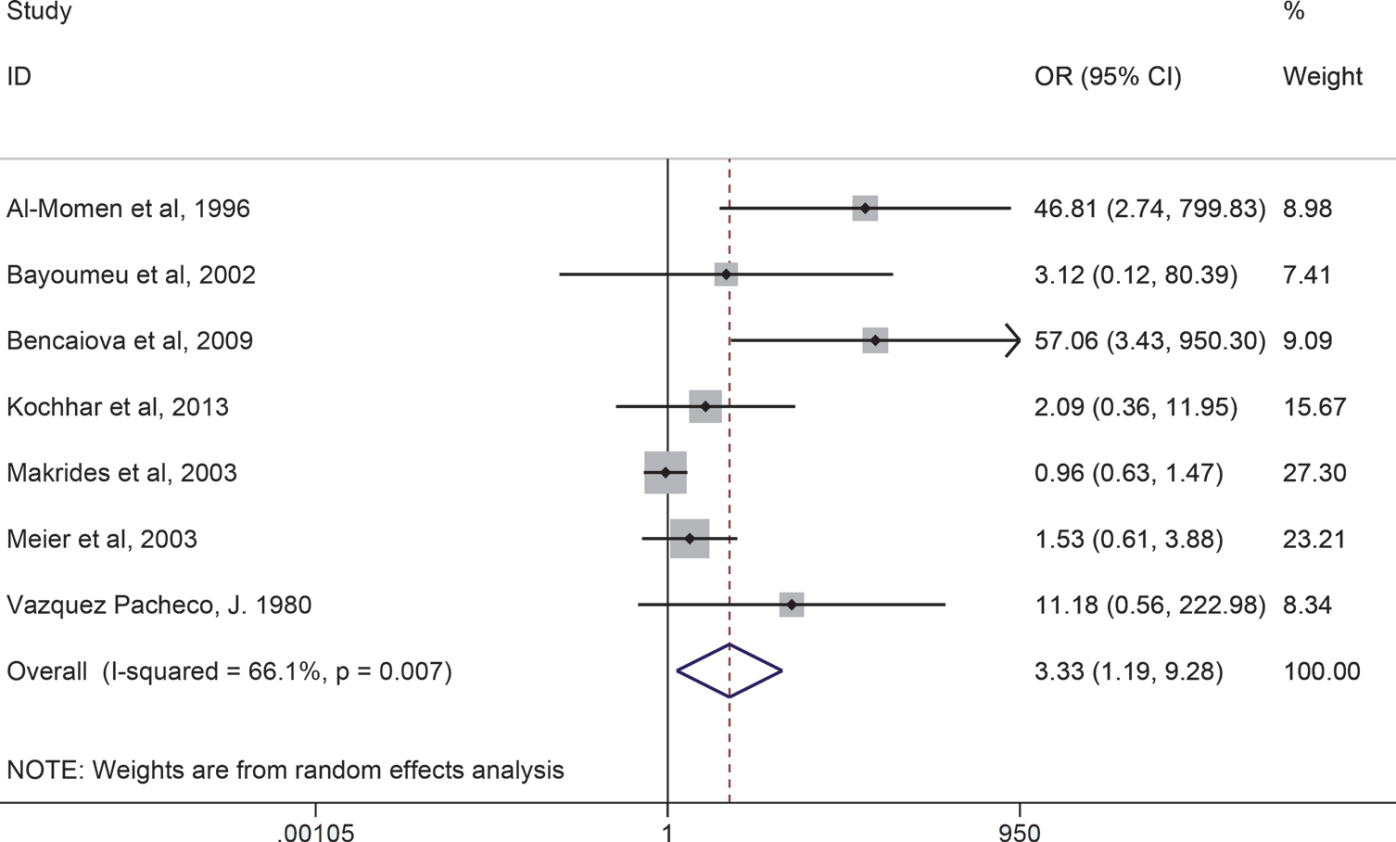
Forest plot for the effect of daily ferrous sulfate supplementation on the incidence of gastrointestinal side-effects in pregnant women. Data for random-effects subgroup meta-analysis are shown (7RCTs, n = 1028). For each study the closed diamond represents the mean estimated effect and the horizontal lines the 95% CI. The grey shaded area surrounding each closed diamond represents the weight of each study in the analysis. Weight was assigned based on (inverse of) the sum of the within-study variance and between study variance. Open diamonds represent the subgroup mean difference and pooled overall mean differences as shown. Test for overall effect: z-score = 2.29; *p* = 0.02. OR, odds ratio; CI, confidence interval.

The subgroup analysis of RCTs in pregnancy generated contrasting results for the placebo-controlled and the IV iron-controlled trials although the number of trials were small (n = 2 and n = 5 respectively, Figs. 2 & 3). Combining data from the 7 RCTs (n = 1028 participants) demonstrated the same detrimental effect of ferrous sulfate with high heterogeneity across studies (OR = 3.33, 95% CI 1.19–9.28, *p* = 0.02, *I* ^*2*^ = 66.1%) (Fig. 4).

### Individual gastrointestinal symptoms reported

Thirty three of the 43 studies reported incidences of individual gastrointestinal symptoms (Table 3). The most commonly reported symptoms were constipation, nausea and diarrhoea. For the 27 studies that reported constipation, the pooled estimate of incidence in the FeSO_4_ arm was 12% [95% CI 10%-15%]. Similarly, for the 30 studies that reported nausea the pooled estimate of incidence in the FeSO_4_ arm was 11% [95% CI 8%-14%] and for the 25 studies that reported diarrhoea the pooled estimate of incidence was 8% [95%CI 6%-11%].

**Table 3:** Individual side-effects reported in the FeSO_4_ group/arm for the studies where this information was available.

First author, year	n	Constipation	Nausea	Diarrhoea	Abdominal pain	Vomiting	Heartburn	Others	Dark stools
**Baykan, 2006** [62]	82	9	1	1	7	1			4
**Cook, 1990** [63]	67	16	15	6	6	0		24 (flatulence)	38
**Davis, 2000** [64]	14	5	5						3
**Fouad, 2013** [65]	20	1	0	3	3			1 (flatulence)	
**Ganzoni, 1974** [66]	90	12	9	21	13		12		
**Gordeuk, 1987** [67]	24	1	11	3	6		2		
**Hallberg, 1966**_1 [24]	175	14	10	10	4		4		
**Hallberg, 1966**_2 [24]	111	11	6	7	8		4		
**Hallberg, 1966**_3 [24]	170	19	5	11	6		3		
**Levy, 1978** [68]	107	27	8	12	13	8	7	24 (flatulence)	
**Maghsudlu, 2008** [69]	185	4	19		5	19			
**Mirrezaie, 2008** [34]	49	1	17		2		5		
**Meier, 2003** [70]	38	9	24	5		13			
**Makrides, 2003** [71]	200	25	58		70	24	136		3
**Pereira,** [40]	10	3	3	2	7		5		8
**Yalcin, 2009** [73]	24	3	1	2	2				4
**Agarwal, 2006** [74]	45	4	2	2					3
**Auerbach, 2004** [75]	43		1						
**Breymann, 2008** [77]	117	8							
**Charytan, 2005** [78]	48	17	5	3		4			
**Henry, 2007** [79]	61	24	16	13	16	12			
**Seid, 2008** [81]	147	16	3		5				
**Tokars, 2010** [82]	91	5	5	5		3	7		
**Van Wyck, 2005** [83]	91	8	5	3		5	1		
**Van Wyck, 2007** [84]	178	20	13	7					
**Van Wyck, 2009** [85]	226	32	27	10		7			
**Kochhar, 2013** [86]	50	4	3	2			2		
**Vazquez Pacheco, 1980** [87]	20		1		3				
**Bayoumeu, 2002** [56]	25			1					
**Kulnigg, 2008** [89]	63		3	4	2				
**Lindgren, 2009** [30]	46		3	9	11	3			2
**Reinisch, 2013** [90]	113	2	1	4	1			1 (abdominal discomfort)	
**Schroder, 2005** [91]	24		5	3	5	5		2 (flatulence)	

Data show for number of subjects reporting each individual symptom.

### Dose-response

**Fig 5:**
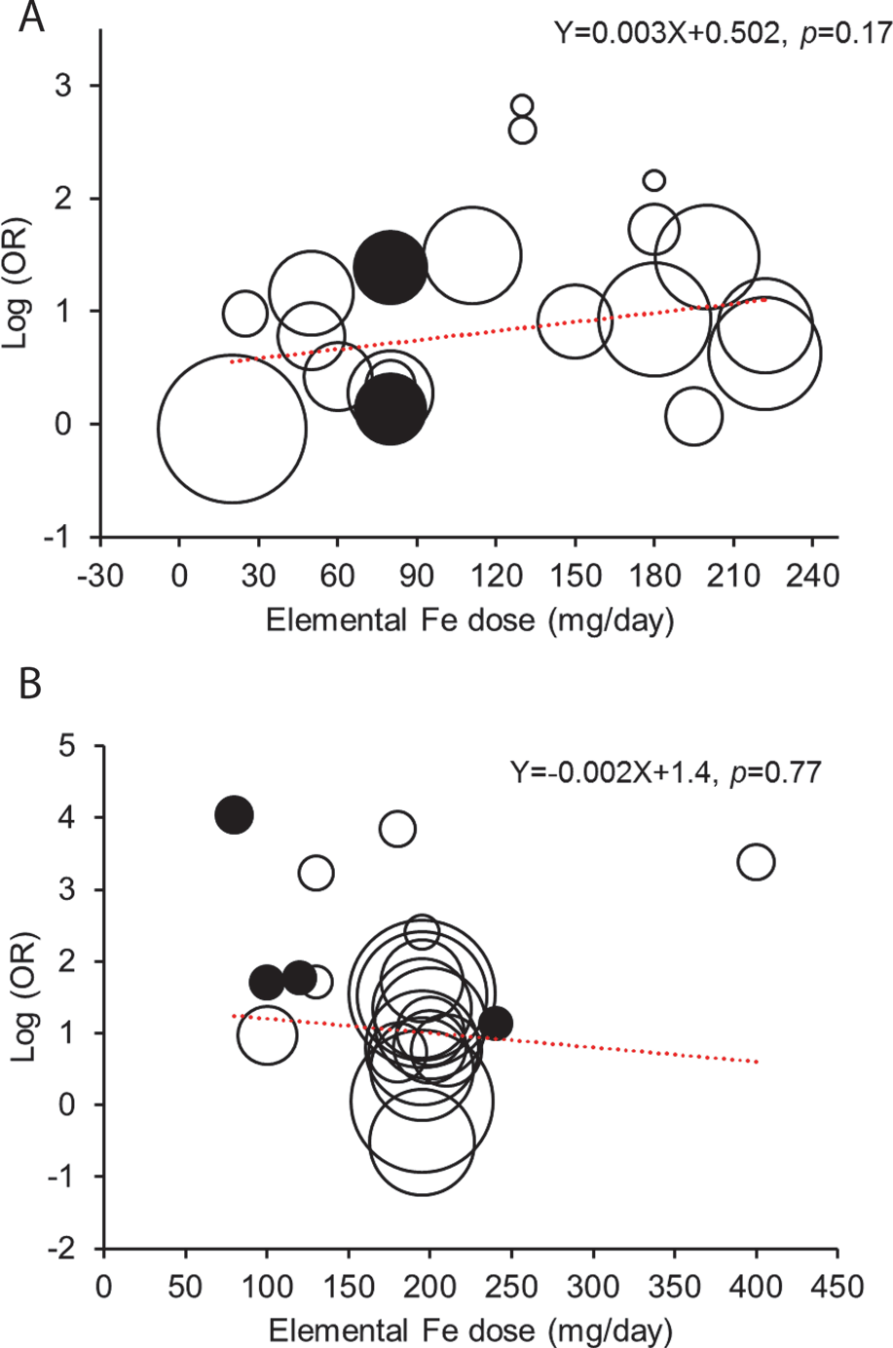
Meta-regression analysis of the association between daily iron dose and the odds ratio of gastrointestinal side-effects. **A,** data from 20 placebo-controlled RCTs (n = 3168); **B,** data from 23 IV iron-controlled RCTs (n = 3663). Individual studies are represented by circles, with the size of the circle being inversely proportional to the variance of the estimated effect (i.e the larger the circle, the more precise the estimated effect). The dotted lines represent the regression line for the analysis. Closed circles, studies with modified release ferrous sulfate; open circles, studies with conventional ferrous sulfate (i.e. not modified-release). All studies used daily posology.

Meta-regression of dose-response effect showed no significant association between dose and gastrointestinal side-effects for both the placebo-controlled [*slope* = 0.003 (95% CI: -0.001–0.007), *p* = 0.17] and the IV iron-controlled [*slope* = -0.002 (95% CI: -0.01–0.01), *p* = 0.77] RCTs (Fig. 5). Indeed, for an iron dose increase of 30 mg, it is estimated that the OR would only change by a factor of 1.08 (95% CI 0.96–1.22) for the placebo comparator trials and 0.96 (95% CI 0.70–1.31) for the IV-iron comparator trials.

## Discussion

WHO considers IDA as one of the most expensive diseases in the world due to lost productivity and the sheer numbers of the population affected (ca. 1 billion) [42]. First-line treatment is oral therapy with ferrous iron salts, but a substantial proportion of patients suffer from gastrointestinal side-effects, resulting in non-adherence and treatment failure [22,23,24,27,28,30,43]. Gastrointestinal symptoms most probably result from a combination of two factors: (i) free radical generation through iron-induced redox cycling in the gut lumen and at the mucosal surface which can promote inflammation [18,44,45,46] and (ii) changes to the microbiota composition or metabolism [17,18,19,47]. Ferrous sulfate remains the most commonly prescribed oral iron therapeutic [10].

We report the first meta-analysis of randomized controlled trials investigating the gastrointestinal side-effects associated with ferrous sulfate supplementation. In our analysis, IV iron was used purely as a comparator arm for gastrointestinal side-effects associated with oral iron for the trials that did not include a placebo arm. The discussion of the side-effects, other than gastrointestinal, associated with IV iron was, therefore, beyond the scope of this analysis.

Significant heterogeneity was observed in both the placebo-controlled studies analysis (*I* ^*2*^ = 53.6%, *p* = 0.002) and the IV iron-controlled studies analysis (*I* ^*2*^ = 41.6%, *p* = 0.02) mainly due to differences in patient population and methodology used to collate gastrointestinal adverse-effects. We have accounted for this heterogeneity by using random-effects modelling [35]. Even though we did not anticipate publication bias to impact the analyses presented here due to the fact that GI adverse-effects was not the primary outcome in most of the studies included in the meta-analysis, we have produced ‘funnel plots’ for visualisation of any asymmetry due to non-publication of studies with negative effects (Figure A in S1 File). The perceived outliers in these plots correspond to studies with zero events in the comparator arms and to one small study with only 10 subjects. The pattern of the remaining studies in the ‘funnel plots’ did not reveal asymmetry and, therefore, there is no indication of publication bias.

Due to the heterogeneity of the study populations in both trial types we have kept the analyses separate overall but there was no change in our findings if the data were combined (*see* Figure C in S1 File). In order to minimize side-effect reporting bias, we have only included controlled trials with a comparator arm that included an intervention, either placebo or intravenous iron, since gastrointestinal symptoms are common even in the general population and are known to be influenced by psychological factors [48,49,50,51]. Both placebo-controlled and IV iron-controlled meta-analysis have shown that ferrous sulfate is associated with increased gastrointestinal side-effects.

Overall, daily ferrous sulfate supplementation was associated with 2.6 times the odds of GI side-effects compared to placebo or IV-iron in participants who were not pregnant and did not have IBD (Figs. 2 & 3). In pregnancy, adherence with ferrous sulfate has been reported as only 70–90% due to adverse-effects [27,28,43]. Two recent Cochrane reviews provide the most comprehensive analysis of oral iron therapy in pregnancy [14,15] and draw on data from 11 trials (n = 4418 participants) reporting side-effects. Pena-Rosas *et al*. have shown that pregnant women receiving oral iron supplements were, overall, more likely than controls (albeit not quite statistically significantly so) to report side effects (25.3% versus 9.91%: RR 2.36; 95% CI 0.96–5.82), particularly at doses ≥ 60 mg of elemental iron [14]. Pregnant women receiving intermittent oral iron supplementation had less side effects (mean RR 0.56; 95% CI 0.37–0.84) than those receiving daily iron supplements [15]. Herein we only considered the 7 eligible trials in pregnant women (n = 1028 participants) that have used oral ferrous sulfate and our findings also show that ferrous sulfate is associated with a significant increase in the incidence of gastrointestinal side-effects (OR = 3.33, *p* = 0.02, Fig. 4), but there is marked heterogeneity in the data.

It is well recognized that inflammatory conditions of the GI tract significantly reduce adherence with oral iron in comparison to the general population, with reports of 52% IBD patients reducing dose or withdrawing from ferrous sulfate treatment due to poor tolerability [29]. Indeed, the four eligible RCTs in IBD patients showed that in this population ferrous sulfate is associated with a significant increase in the incidence of GI side-effects (OR = 3.14, *p* = 0.008, Fig. 3) in comparison to intravenous iron. However, in the IV iron-controlled studies all subjects were considerably ill with moderate-severe anemia and/or other underlying conditions and, therefore, the comparison with an ‘otherwise healthy’ population group was not possible.

Even following considerable efforts through local and national libraries we could not obtain the full text for 88 studies (Table C in S1 File). The vast majority of these papers were old and not available in English translation, and for many there was no abstract available. Nonetheless, based on a rate of inclusion of 2% from full-text (Fig. 1), we estimate that only 1–2 studies would have been eligible for inclusion from the 88 references that were not obtained. Even though this remains a minor weakness of the present meta-analysis, it would be surprising if data from these few anticipated, additional eligible trials would have changed our findings.

Even though we strived to include in this meta-analysis only robustly designed trials, one limitation remains in relation to blinding of the treatments. First, IV versus oral iron trials are difficult to blind due to the nature of the different interventions. None of the IV iron-controlled studies were blinded and, therefore, we have judged this to represent a high risk of bias in relation to reporting of the main outcome of the present analysis (i.e. GI adverse-effects) (Table A in S1 File). However, we do acknowledge that for the vast majority of the studies the primary outcome measures were biochemical parameters (e.g. hemoglobin) and this is unlikely to have been impacted to the same extent by the lack of blinding.

Secondly, the placebo-controlled trials were not *truly* blind studies as blackened stools are commonly reported with oral iron [52,53] and in none of the trials included in our meta-analysis was use of a ‘stool darkener’ reported for the placebo arm. Therefore, patients would mostly have been aware when they were taking oral iron and perception of gastrointestinal symptoms could have been altered in a manner that even robust meta-analysis cannot account for. To support the double-blind design in future placebo-controlled clinical trials investigating gastrointestinal symptoms with oral iron it would be preferable to use a stool darkener

It is generally considered that (i) doses ≤ 50–60 mg iron/day generate less side-effects than higher doses and that (ii) iron given in controlled release formulations is better tolerated [27,54,55]. However, our meta-regression analysis shows that there is no statistically significant dose-response effect or threshold whether considered as amount of iron per day (Fig. 5, Figure D-A in S1 File) or per dose (Figure D-B in S1 File). A limitation of this analysis, however, is that data are not uniformly spread across iron dosage, particularly for the intravenous iron-controlled trials that mostly used ~200mg Fe/day (see Fig. 5B). Nonetheless, if there is a benefit in terms of lower side-effects with lower doses of ferrous sulfate then the threshold appears very low (i.e. ≤ 20 mg iron per dose or per day, Figure D in S1 File).

A recent review of 111 studies (10695 participants) with different oral iron preparations has suggested that slow-release ferrous sulfate is better tolerated than regular gastric release ferrous iron salts [11]. This review has included all types of studies ranging from observational to RCT. In contrast, we restricted the work here to meta-analysis of RCTs with robust trial design which still included data from 6831 participants. In this case, subgroup analysis of the 6 included studies that used modified-release ferrous sulfate [56,57,58,59,60,61] in fact shows an OR = 3.60 (95% CI 1.32–9.87, *p* = 0.01, *I* ^*2*^ = 46.2%) compared to OR = 2.53 (95% CI 1.99–3.21, *p*<0.0001, *I* ^*2*^ = 49.6%) for the 37 studies that have used conventional delivery of oral ferrous sulfate (*see* Figure C in S1 File). Four of the studies with modified-release ferrous sulfate [56,57,58,61] reported zero GI side-effects in the IV iron arm so these data should be interpreted with caution. Nonetheless, our findings do not support the idea that modified-release oral ferrous sulfate markedly modifies its side-effects.

## Conclusions

In summary, our analyses show that: (i) ferrous sulfate causes significant gastrointestinal side-effects in adults in all the population groups investigated, with the caveat of potential biases associated with study blinding that are inherent to interventions with oral iron, as discussed above; (ii) the OR of side-effects in IBD is higher than in non-IBD and non-pregnant participants but overall numbers were small and significance not established; (iii) the pregnancy subgroup analysis revealed considerable heterogeneity; (iv) there is no evidence for dose effect; (v) there is no evidence that modified-release ferrous sulfate causes less side-effects than conventional gastric release ferrous sulfate.

## Supporting Information

S1 PRISMA Checklist PRISMA checklist. (DOCX)

S1 File **Figure A**, Funnel plots of effect of daily ferrous sulfate supplementation on the incidence of gastrointestinal side-effects against standard error. **Figure B,** Forest plot for the effect of daily ferrous sulfate supplementation on the incidence of gastrointestinal side-effects in IV iron-controlled RCTs. **Figure C,** Forest plot for the effect of daily ferrous sulfate supplementation on the incidence of gastrointestinal side-effects. **Figure D,** Meta-regression analysis of the association between iron dosage and the odds ratio of gastrointestinal side-effects. **Methods A. Table A,** Assessment of ‘risk of bias’ according to the Cochrane Collaboration’s tool. **Table B,** Mean hemoglobin increase (g/dl) reported in IV iron-controlled RCTs (n = 3267). **Table C,** References identified in the systematic search for which full-text could not be obtained.
(PDF)
