# Supplementary material for: Ferrous Sulfate Supplementation Causes Significant Gastrointestinal Side-Effects in Adults: A Systematic Review and Meta-Analysis
Source: PLoS One. 2015 Feb 20;10(2):e0117383. doi: 10.1371/journal.pone.0117383 (PMC4336293; doi:10.1371/journal.pone.0117383)
Supplement: S1 File — Figure B, Forest plot for the effect of daily ferrous sulfate supplementation on the incidence of gastrointestinal side-effects in IV iron-controlled RCTs. Figure C, Forest plot for the effect of daily ferrous sulfate supplementation on the incidence of gastrointestinal side-effects. Figure D, Meta-regression analysis of the association between iron dosage and the odds ratio of gastrointestinal side-effects. Methods A. Table A, Assessment of ‘risk of bias’ according to the Cochrane Collaboration’s tool. Table B, Mean hemoglobin increase (g/dl) reported in IV iron-controlled RCTs (n = 3267). Table C, References identified in the systematic search for which full-text could not be obtained. (PDF) [file pone.0117383.s002.pdf]

Methods.

Full electronic search strategy

The PubMed search syntax listed below served as the basis for all search strategies. This search strategy was then adapted to the other electronic databases searched.

((*iron* [MeSH terms] OR ferrous [MeSH terms]) OR (*ferrous sulphate* [All fields] OR *ferrous sulfate* [All fields] OR *iron sulphate* [All fields] OR *iron sulphate* [All fields] OR *oral iron* [All fields] OR iron supplementation [All fields] OR iron supplement [All fields] OR intravenous iron [All fields])) AND ((*trial* [MeSH terms] OR *study*[MeSH terms]) OR (*randomised controlled trial* [All fields] OR *randomized controlled trial* [All fields] OR *controlled trial* [All fields] OR *controlled study* [All fields] OR *human study* [All fields] OR *intervention study* [All fields] OR *randomised study* [All fields] OR *randomized study* [All fields] OR *randomised trial* [All fields] OR *randomized trial* [All fields])).

Online Supplementary Material

Table A. Assessment of 'risk of bias' according to the Cochrane Collaboration's tool (1).

Placebo-controlled trials:

First Author, Year	n total	n $FeSO_4$	n placebo	comments on missing data	random sequence generation	allocation concealment	blinding of participants, personnel and outcome assessors	incomplete outcome data	selective outcome reporting	other sources of bias	Bias from the way side-effects were recorded	
Baykan, 2006	168	82	86	numbers as randomised	high risk	high risk	high risk	low risk	unclear	unclear	unclear	telephone interview
Cook, 1990	133	67	66	numbers as randomised	low risk	low risk	low risk	low risk	unclear	unclear	low risk	symptoms questionnaire
Davis, 2000	28	14	14	numbers as randomised	low risk	unclear	low risk	low risk	unclear	unclear	unclear	telephone interview
Fouad, 2013	40	20	20	no missing data	low risk	low risk	low risk	low risk	unclear	unclear	low risk	symptoms questionnaire
Ganzoni, 1974	90	90	90	numbers as randomised	unclear	unclear	low risk	low risk	unclear	unclear	unclear	face to face interview
Gordeuk, 1987	47	24	23	numbers where adverse effects were assessed	unclear	unclear	low risk	low risk	unclear	unclear	low risk	questionnaire
Hallberg 1966, 1	344	175	169	numbers where adverse effects were assessed	unclear	unclear	low risk	low risk	unclear	unclear	low risk	questionnaire
Hallberg 1966, 2	226	111	115	numbers where adverse effects were assessed	unclear	unclear	low risk	low risk	unclear	unclear	low risk	questionnaire
Hallberg 1966, 3	347	170	177	numbers where adverse effects were assessed	unclear	unclear	low risk	low risk	unclear	unclear	low risk	questionnaire
Levy, 1978	107	107	107	numbers where adverse effects were assessed	low risk	unclear	low risk	low risk	unclear	unclear	low risk	questionnaire
Maghsudlu, 2008	367	185	182	numbers where adverse effects were assessed	unclear	unclear	unclear	low risk	unclear	unclear	unclear	face to face interview
Mirrezaie, 2008	95	49	46	numbers as randomised	low risk	unclear	low risk	low risk	unclear	unclear	unclear	telephone interview
Makrides, 2003	393	200	193	numbers where adverse effects were assessed	low risk	low risk	low risk	low risk	unclear	unclear	low risk	structured telephone questionnaire
Meier, 2003	74	38	36	numbers as randomised	unclear	unclear	low risk	low risk	unclear	unclear	low risk	telephone interview
Pereira, 2014	20	10	10	no missing data	low risk	unclear	low risk	low risk	unclear	unclear	low risk	symptoms questionnaire
Sutton, 2004	72	35	37	numbers as randomised	low risk	low risk	low risk	low risk	unclear	unclear	low risk	questionnaire
Tuomainen, 1999	30	15	15	numbers where adverse effects were assessed	unclear	unclear	low risk	low risk	unclear	unclear	unclear	spontaneous reporting
Vaucher, 2012	198	102	96	numbers as randomised	low risk	low risk	low risk	low risk	low risk	unclear	unclear	interview
Yalcin, 2009	47	24	23	numbers as randomised	high risk	high risk	unclear	low risk	unclear	low risk	unclear	telephone interview
Waldvogel, 2012	145	74	71	numbers where adverse effects were assessed	low risk	low risk	low risk	low risk	unclear	unclear	unclear	interview

Table A continued. Assessment of 'risk of bias' according to the Cochrane Collaboration's tool (1)

IV iron-controlled trials:

First Author, Year	n total	n $FeSO_4$	n placebo	comments on missing data	random sequence generation	allocation concealment	blinding of participants, personnel and outcome assessors[(1)]	incomplete outcome data	selective outcome reporting	other sources of bias	Bias from the way side-effects were recorded	
Agarwal, 2006	89	45	44	numbers as randomised	low risk	low risk	high risk	low risk	unclear	unclear	unclear	spontaneous reporting
Auerbach, 2004	121	43	78	numbers as randomised	unclear	unclear	high risk	low risk	unclear	unclear	unclear	telephone interview
Bhandal, 2006	43	21	22	numbers with data (1 less than randomised)	low risk	low risk	high risk	low risk	unclear	unclear	unclear	spontaneous reporting
Breymann, 2008	344	117	227	numbers where adverse effects were assessed	unclear	unclear	high risk	low risk	low risk	unclear	unclear	spontaneous reporting
Charytan, 2005	96	48	48	numbers where adverse effects were assessed	unclear	unclear	high risk	low risk	low risk	unclear	unclear	spontaneous reporting
Henry, 2007	124	61	63	numbers where adverse effects were assessed	unclear	low risk	high risk	low risk	unclear	unclear	unclear	face to face interview
Mudge, 2012	102	51	51	as randomised	low risk	low risk	high risk	low risk	unclear	unclear	unclear	face to face interview
Seid, 2008	289	147	142	numbers where adverse effects were assessed	low risk	low risk	high risk	low risk	low risk	unclear	unclear	spontaneous reporting
Strickland, 1977	20	20	20	numbers as randomised	low risk	unclear	high risk	unclear	unclear	unclear	low risk	questionnaire
Tokars, 2010	182	91	91	numbers as randomised	unclear	unclear	high risk	low risk	unclear	unclear	unclear	unclear
Van Wyck, 2005	182	91	91	numbers where adverse effects were assessed	unclear	unclear	high risk	low risk	unclear	unclear	unclear	spontaneous reporting
Van Wyck, 2007	352	178	174	numbers where adverse effects were assessed	low risk	low risk	high risk	low risk	unclear	unclear	unclear	spontaneous reporting
Van Wyck, 2009	456	226	230	numbers where adverse effects were assessed	low risk	low risk	high risk	low risk	unclear	unclear	unclear	spontaneous reporting
Vazquez Pacheco, 1980	40	20	20	numbers as randomised	unclear	unclear	high risk	low risk	unclear	unclear	unclear	unclear
Al Momen, 1996	111	59	52	numbers as randomised	unclear	unclear	high risk	low risk	unclear	unclear	unclear	face to face interview
Bayoumeu, 2002	50	25	25	numbers as randomised	low risk	unclear	high risk	low risk	unclear	unclear	unclear	face to face interview
Bencaiova, 2009	260	130	130	numbers as randomised	low risk	low risk	high risk	unclear	unclear	unclear	unclear	unclear
Kulnigg, 2008	200	63	137	numbers as randomised	low risk	low risk	high risk	low risk	low risk	unclear	unclear	face to face interview
Lindgren, 2009	91	46	45	numbers as randomised	low risk	low risk	high risk	unclear	unclear	unclear	unclear	spontaneous reporting
Schroder, 2005	46	24	22	numbers as randomised	low risk	unclear	high risk	low risk	unclear	unclear	unclear	spontaneous reporting
Kochhar, 2013	100	50	50	numbers as randomised	low risk	low risk	high risk	low risk	low risk	unclear	unclear	face to face interview
Reinisch, 2013	338	109	223	numbers where adverse effects were assessed	low risk	low risk	high risk	low risk	low risk	unclear	low risk	questionnaire
Guerra Merino, 2012	13	7	6	numbers as randomised	low risk	low risk	high risk	low risk	low risk	unclear	unclear	spontaneous reporting

(1) note that these IV iron-controlled studies were not blinded and we have judge them as having high risk of bias specifically for the reporting of adverse effects due to the nature of the intereventions (i.e. intravenous versus oral).

1. Higgins JP, Altman DG, Gotzsche PC, Juni P, Moher D, Oxman AD, et al. The Cochrane Collaboration's tool for assessing risk of bias in randomised trials. BMJ. 2011;343:d5928.

Table B. Mean hemoglobin increase (g/dl) reported in IV iron-controlled RCTs (n=3267).

First author, year	$FeSO_4$ arm	IV-iron arm
**Agarwal, 2006**	0.20	0.40
**Auerbach, 2004**	1.50	2.40
**Bhandal, 2006**	3.70	4.20
**Breymann, 2008**	3.13	3.44
**Charytan, 2005**	0.70	1.00
**Henry, 2007**	1.60	2.40
**Seid, 2008**	3.40	4.00
**Tokars, 2010**	0.8	1.1
**Van Wyck, 2005**	0.40	0.70
**Van Wyck, 2007**	3.30	4.10
**Van Wyck, 2009**	2.30	3.10
**Vazquez Pacheco, 1980**	0.74	3.94
**Al-Momen, 1996**	3.48	5.27
**Bayoumeu, 2002**	0.30	1.51
**Kulnigg, 2008**	3.00	3.60
**Lindgren, 2009**	2.22	2.41
**Schroder, 2005**	2.10	2.50
**Kochhar, 2013**	3.1	5.1
**Reinisch, 2013**	2.98	2.59
**Guerra Merino, 2012**	4.2	3.5

Online Supplementary Material

Table C. References identified in the systematic search for which full-text could not be obtained.

1.	Kreutzkamp B. Postpartum iron deficiency anemia: Intravenous ferric carboxymaltose is superior to oral iron substitution [German] Postpartale eisenmangelanamie: Eisen-carboxymaltose i. v. ist oraler fe-substitution uberlegen. Medizinische Monatsschrift fur Pharmazeuten. 2009;32(7):273-4.
2.	Jaleel A, UrRahman MA. Daily iron supplementation versus intermittent dose. Saudi Medical Journal. 2004;25(8):1124-5.
3.	Carre N, Eono P, Kouakou K, Duponchel JL, Marquis M, Zahui KH. Iron supplementation associated with malaria prevention among pregnant women in Abidjan. Rev Epidemiol Sante Publique (Revue d'Epidemiologie et de Sante Publique). 2003;51(1 (Pt 1)):31-8.
4.	Makrides M, Crowther CA, Gibson RA, Gibson RS, Skeaff CM. Low-dose iron supplements in pregnancy prevent iron deficiency at the end of pregnancy and during the post-partum period: The results of a randomised controlled trial. Asia Pacific Journal of Clinical Nutrition. 2002;11(SUPPL.).
5.	Basile JN. ACE inhibitor-associated cough lessened with iron supplementation. Journal of clinical hypertension (Greenwich, Conn). 2002;4(1):49-50.
6.	Kaul S, LaCreta FP, Kollia GD, Duncan G, Randall DM, Grasela DM. Pharmacokinetics and safety of gatifloxacin in combination with ferrous sulfate or calcium carbonate in healthy volunteers. Journal of Infectious Disease Pharmacotherapy. 2001;5(1):37-55.
7.	Continuous, rather than intermittent, IV iron administration is recommended. Erythropoiesis: New Dimensions in the Treatment of Anaemia. 2000;10(2):41-2.
8.	Goya N, Toda F, Nishino S, Tokumoto T, Iguchi Y, Kobayashi H, et al. Autotransfusion supported by erythropoietin therapy in transurethral resection of the prostate. Scandinavian Journal of Urology and Nephrology. 1998;32(6):383-7.
9.	Fudin R, Jaichenko J, Shostak A, Bennett M, Gotloib L. Correction of uremic iron deficiency anemia in hemodialyzed patients: a prospective study. Nephron. 1998;79(3):299-305.
10.	Bowden JA. Relative absorption of iron and magnesium from sulfate salts, amino acid chelates complexed and/or mixed with vegetables, and taste-free supplements [M.S.]. United States -- Utah: Utah State University; 1997.
11.	Viteri FE, Ali F, Tujague J. Weekly iron supplementation of fertile-age women achieves a progressive increment in serum ferritin. FASEB Journal. 1996;10(3):A292.
12.	Svara F, Sulkova S, Kvasnicka J, Polakovic V. Iron supplementation during erythropoietin treatment of haemodialyzed patients. Vnitrni Lekarstvi. 1996.
13.	Breymann C, Zimmermann R, Huch R, Huch A. Use of recombinant human erythropoietin in combination with parenteral iron in the treatment of postpartum anaemia. European Journal of Clinical Investigation. 1996;26(2):123-30.
14.	Zimmermann R, Breymann C, Breymann C Fau - Richter C, Richter C Fau - Huch R, Huch R Fau - Huch A, A H. rhEPO treatment of postpartum anemia. J Perinat Med (Journal of Perinatal Medicine). 1995;23(1-2):111-7.
15.	Gedik Y, Erduran E, Mocan H, Aynaci FM, Okten A. The efficacy of ferrous sulfate and ferric polymaltose in therapy of iron deficiency anemia. Annals of Medical Sciences. 1995;4(2):143-4.
16.	Frondaroli F, Di Leonardo A, Tella D, Khalig JG. Epidemiology of sideropenic anemias and beta-thalassemia in pregnancy in the city of Chieti (Italian) EPIDEMIOLOGIA DELL'ANEMIA SIDEROPENICA E DELLA Î²-TALASSEMIA IN GRAVIDANZA NEL TERRITORIO DI CHIETI. Minerva Ginecologica. 1994;46(10):557-60.
17.	Crosby L, Palarski VA, Cottington E, Cmolik B. Iron supplementation for acute blood loss anemia after coronary artery bypass surgery: A randomized, placebo-controlled study. Heart and Lung: Journal of Critical Care. 1994;23(6):493-9.

18. Del Cura J, Maside C, Rus A, Borque I. Reference values of serum iron, ferritin and transferrrin in pregnancy. Effect of intake of iron supplementation (Spanish) VALORES DE REFERENCIA DE HIERRO, FERRITINA Y TRANSFERRINA SERICAS EN LA GESTACION. EFECTO DE LA INGESTA DE SUPLEMENTOS DE HIERRO. Progresos en Obstetrica y Ginecoligia. 1993;36(5):233-8.

19. Arnaud J, Prual A, Preziosi P, Cherouvrier F, Favier A, Galan P, et al. Effect of iron supplementation during pregnancy on trace element (Cu, Se, Zn) concentrations in serum and breast milk from Nigerian women. Ann Nutr Metab (Annals of Nutrition and Metabolism). 1993;37(5):262-71.

20. Dodd NS, Sheela TS, Sharma UK. Effect of different levels of iron supplementation on the iron status and physical work capacity of anaemic Indian women. Indian Journal of Medical Sciences. 1992;46(2):33-42.

21. Agarwal KN, Agarwal DK, Mishra KP. Impact of anaemia prophylaxis in pregnancy on maternal haemoglobin, serum ferritin & birth weight. Indian Journal of Medical Research. 1991;94:277-80.

22. Yoshida T, Udo M, Chida M, Ichioka M, Makiguchi K. Dietary iron supplement during severe physical training in competitive female distance runners. Sports Training, Medicine and Rehabilitation. 1990;1(4):279-85.

23. Jacquot C, Berthelot JM, Chiappini-Judith D, Ferragu-Haguet M, Lefebvre A, Masselot JP, et al. [Treatment of anemia in chronic hemodialysis patients with recombinant human erythropoietin: long-term results in 15 patients] French. Nephrologie. 1990;11(1):11-6.

24. Giordano N, Borghi C, Sancasciani S, Morozzi G, Fioravanti A, Magaro L, et al. Efficacy and safety of an iron chelating agent, deferoxamine, in patients with rheumatoid arthritis and hyposideremic anemia. Current Therapeutic Research - Clinical and Experimental. 1990;48(6):959-68.

25. Di G, D'Antonio S, Ravallese F, Di D. Iron therapy in the hypochromic anemia. Controlled clinical trial [Italian] TERAPIA MARZIALE NELL'ANEMIA IPOCROMICA. STUDIO CLINICO CONTROLLATO. Terapia Moderna. 1990;4(4):251-3.

26. Daniels Jr EH, Ente G, Freed N, Levy B, Lovett WC, Knipfer MA, et al. Hemoglobin response to two modified-release iron formulations in patients with iron deficiency anemia: An evaluator-blind trial. Today's Therapeutic Trends. 1989;7(2):11-9.

27. Bloxam DL, Williams NR, Waskett RJ, Pattinson-Green PM, Morarji Y, Stewart SG. Maternal zinc during oral iron supplementation in pregnancy: a preliminary study. Clinical Science. 1989;76(1):59-65.

28. Pejtsik B, Pacsa S, Pereszlenyi L. Treatment of gestational iron deficiency disease with tardyferon; monitoring the effectivity of treatment. Therapia Hungarica. 1988;36(1):25-8.

29. Munoz Carratala M, Moya Benavent M, De la Cruz Amoros V, Herrero Galiana A, Juste Ruiz M. Factors modifying the efficacy of the treatment of iron-deficiency anemia with ferrous sulphate. Revista Espanola de Pediatria. 1988;44(262):381-4.

30. Farago E. Examination of Tardyferon depot coated tablet in patients treated by general practitioners. Therapia Hungarica. 1988;36(2):81-5.

31. Matter M, Stittfall T, Graves J, Myburgh K, Adams B, Jacobs P, et al. The effect of iron and folate therapy on maximal exercise performance in female marathon runners with iron and folate deficiency. Clin Sci. 1987;72(4):415-22.

32. AIa Mb. [Are any drug admixtures except iron preparations required in the treatment of iron deficiency anemia?]. Ter Arkh (Terapevticheskii Arkhiv). 1987;59(6):119-24.

33. Linpisarn S, Kunachiwa W, Laokuldilok T, Laokuldilok J, Keawvichit R, Kulapongs P. Iron status and the effect of iron supplementation in Thai male blood donors in northern Thailand. Southeast Asian J Trop Med Public Health (THE SOUTHEAST ASIAN JOURNAL OF TROPICAL MEDICINE AND PUBLIC HEALTH). 1986;17(2):177-83.

34. Fleming AF, Ghatoura GBS, Harrison KA. The prevention of anaemia in pregnancy in primigravidae in the guinea savanna of Nigeria. Annals of Tropical Medicine and Parasitology. 1986;80(2):211-33.

35. Rybo E, Bengtsson C, Hallberg L, Oden A. Effect of iron supplementation to women with iron deficiency. Scandinavian journal of haematology Supplementum. 1985 1985;34(S43):103-14.

36. Wallenburg HCS, Van HG. Effect of oral iron supplementation during pregnancy on maternal and fetal iron status. Journal of Perinatal Medicine. 1984;12(1):7-12.

37. Harju E, Pakarinen A. The effect of iron treatment on serum ferritin concentrations and bone marrow stainable iron in iron deficient out-patients with gastritis, gastric ulcer and duodenal ulcer. J Int Med Res (The Journal of International Medical Research). 1984;12(1):56-8.

38. Heyder N, Waldherr A, Hartwich G. Is ferric ion absorbed? Absorption of ferrous and ferric ions in healthy subjects and anemic patients (German) Wird 3wertiges Eisen resorbiert? Resorptionsverhalten von 2- und 3wertigem Eisen bei Gesunden und anämischen Patienten. Fortschritte der Medizin. 1982;100(26):1259-61.

39. Gringras M. A comparison of two combined iron-folic acid preparations in the prevention of anaemia in pregnancy. Journal of International Medical Research. 1982;10(4):268-70.

40. Ravetta A. Iron blood level in the elderly LA SIDEREMIA NELL'ANZIANO. European Review for Medical and Pharmacological Sciences. 1981;3(3):217-22.

41. Nietfeld C. Iron therapy of anemia in pregnancy and puerperium EISEN-THERAPIE DER ANAMIE IN DER SCHWANGERSCHAFT UND IM WOCHENBETT. Fortschritte der Medizin. 1981;99(34):1370-2.

42. Charoenlarp P. Iron supplementation studies. Experimental design and interpretation of results. Progress in clinical and biological research. 1981;77:355-61.

43. Blunden RW, Casey GJ, Giorgio B. The effect of normal and high dose iron supplementation on serum ferritin levels during pregnancy. Journal of Obstetrics and Gynaecology. 1981;2(1):20-4.

44. Ogunbode O, Damole IO, Oluboyede OA. Iron supplement during pregnancy using three different iron regimens. Curr Ther Res, Clin Exp (Current Therapeutic Research, Clinical and Experimental). 1980;27(1):75-80.

45. Kiire C, Nantulya V, Otim M. The treatment of severe hookworm anaemia by a sustained slow-release ferrous sulphate preparation, Plexafer. East African medical journal. 1980;57(3):218-22.

46. Heinrich HC, Fuhr J. [Resorption of oral iron preparations]. [German] Experimenteller Beitrag zur Resorption oraler Eisengaben. Die Medizinische Welt. 1980;31(29-30):1104-5.

47. Fernandez X, Young B, Lavin P, Baeza R, Seaman V. The IUD and anemia: a study of hematocrit. Contraceptive Delivery Systems. 1980;1(1):49-53.

48. Abdulkadyrov KM, Belyakova TA, Andrianova IG. Treatment of iron deficiency anemia and prevention of its relapses by Soviet iron-containing drugs. Klinicheskaya Meditsina. 1980;58(4):61-7.

49. Szabo A, Krivacsy G, Haness J, Pinter S. Ferrous sulfate plus sodium dioctyl sulfosuccinate in treatment of pregnancy anemia (German) CONFERON IN DER BEHANDLUNG DER SCHWANGERSCHAFTSANAMIE. Therapia Hungarica. 1979;27(4):170-3.

50. Heinrich HC. Comparison of 2 drugs for oral iron therapy [German] Vergleichende Prufung zweier Praparate zur oralen Eisentherapie. Die Medizinische Welt. 1979;30(51-52).

51. Gerlach E. Effective iron therapy on a new basis [EFFEKTVOLLE EISENTHERAPIE MIT NEUEM KONZEPT]. Zeitschrift fur Allgemeinmedizin. 1979;55(4):255-8.

52. Canto TE, Cetina JA, Reyes A, Viveros A. Hematologic effects of ferrous sulfate combined with folic acid and vitamin B12 in pregnancy with anemia (Spanish) EFECTOS HEMATOLOGICOS DEL SULFATO FERROSO ASOCIADO CON ACIDO FOLICO Y VITAMINA B12, EN EMBARAZADAS CON ANEMIA. Investigacion Medica Internacional. 1979;6(2):145-51.

53. Weippl G. Treatment of iron deficiency anemia with ferrosulfate serin complex THERAPIE DER EISENMANGELANAMIE ERFAHRUNGEN MIT EINEM SERIN FERROSULFAT KOMPLEX. Padiatr Padol (Padiatrie und Padologie). 1978;13(1):91-6.

54. Stembera Z, Srpova M. The treatment of anaemia by Ferro-gradumet in pregnancy (Czech). Cesko-Slovenska Gynekologie. 1978;43(8):595-8.

55. Mueller R. Clinical trial with measurement of iron absorption from a mixture of ferrous sulfate and ascorbic acid [STUDIE ZUR KLINISCHEN PRUFUNG DER ORALEN EISENRESORPTION VON CE-FERRO FORTE]. Materia Medica Nordmark. 1978;30(3-4):75-84.

56. Metzger KH. A new oral iron preparation with a new concept. (German) ORALES EISEN THERAPEUTIKUM MIT NEUEM KONZEPT. Fortschritte de Medezin. 1978;96(14):779-82.

57. Lehmann H, Kuhl ED. Comparative testing of 2 preparations for oral iron therapy [German] Vergleichende Prufung zweier Praparate zur oralen Eisentherapie. Die Medizinische Welt. 1978;29(15):632-4.

58. Halliday JA, Taft LI, Russo AM. Serum ferritin in pregnancy: A new look at iron stores and the effect of iron supplementation. Australian and New Zealand Journal of Medicine. 1978;8(6):675.

59. Bernat I, Magyari J, Bernat I, Mihalyi L. Plasma iron concentrations after oral administration of various iron preparations. Transzfuzio. 1978;12(1):5-11.

60. Afifi AM. Plexafer-F in the management of latent iron deficiency in pregnancy. J Int Med Res (The Journal of International Medical Research). 1978;6(1):34-40.

61. Wiersinga A, Amatayakul K, Kulapongs P, Olson RE. Iron deficiency anemia in pregnancy: Comparison of total dose infusion using iron dextran complex to oral iron therapy. Journal of the Medical Association of Thailand. 1977;60(12):595-609.

62. Svanberg B, Rybo G. Side-effects and placental function in six pregnant women treated with Ferastral. Scandinavian journal of haematology Supplementum. 1977;19(32):355-62.

63. Strickland ID, Chaput de Saintonge DM, Boulton FE, Francis B, Roubikova J, Waters JI. The therapeutic equivalence of oral and intravenous iron in renal dialysis patients. Clin Nephrol. 1977;7(2):55-7.

64. Savin MA. A practical approach to the treatment of iron deficiency. Rational Drug Therapy. 1977;11(9):1-6.

65. Popko J, Slowikowska Koniecko J, Bogdanikowa B. Tardyferon preparation in the treatment of hypochromic anemia (Polish). Polski Tygodnik Lekarski. 1977;32(1):21-2.

66. Ogunbode O, Oluboyede OA, Ayeni O. The treatment of iron-deficiency anaemia with a new intramuscular iron preparation (Ferastral). Scand J Haematol Suppl (SCANDINAVIAN JOURNAL HAEMATOLOGY SUPPLEMENTUM). 1977;32:364-71.

67. De Arreola GA, Nevarez Najera A, Ganot Rodriguez J, Rivera Damm R. The use of a new ferrous sulfate preparation in the treatment of iron deficiency anemia (Spanish) EL USO DE UNA NUEVA PREPARACION DE SULFATO FERROSO EN EL TRATAMIENTO DE LAS ANEMIAS POR DEFICIENCIA DE HIERRO. Medicina Revista Mexicana. 1977;57(1216):17-25.

68. Belyakova TA, Smirnova ZA, Yudovskaya VM. Functional condition of the stomach of patients with hypoferrous anemia given ferrous preparations as prophylaxis (Russian). Terapevticheskii Arkhiv. 1977;49(1):52-6.

69. Rossi E, Serventi G, Venturini A, Arisi L, Bignardi L, Garini G, et al. [Oral and i.v. iron therapy in haemodialysis patients (author's transl)]. Ateneo Parmense Acta Biomed. 1976;47(1):33-46.

70. Todorovic M, Cvrije V. Study on the therapeutic and side-effects of an iron preparation with delayed action in the treatment of sideropenic anemia. Lijecnicki Vjesnik. 1975;97(11):616-8.

71. Llaca Rodriguez V, Fernandez Alba J. Assessment of the tolerance of a compound with ferrous sulphate administered by oral route during pregnancy (Spanish) EVALUACION DE LA TOLERANCIA DE UN PREPARADO A BASE DE SULFATO FERROSO ADMINISTRADO POR VIA ORAL DURANTE LA GESTACION. Medicina. 1975;55(1203):394-7.

72. Feustel H, Schricker KT, Bayerschmidt HP. Effect of oral iron therapy in blood donors on diverse blood parameters. Med Welt (Die Medizinische Welt). 1975;26(5):186-8.

73. Bokarev IN, Andreeva VK, Astashenko NA. Comparative assessment of the efficacy of peroral treatment of patients with chronic posthemorrhagic anemia with iron containing preparations (Russian). Klinicheskaya Meditsina. 1975;53(11):110-3.

74. Weswig PH, Winkler W, Jr. Iron supplementation and hematological data of competitive swimmers. J Sports Med Phys Fitness (The Journal of Sports Medicine and Physical Fitness). 1974;14(2):112-9.

75. Strickland ID, Chaput De Saintonge DM, Boulton FE, Brain AJ, Goodwin FJ, Marsh FP, et al. A trial of oral iron in dialysis patients. Clinical Nephrology. 1974;2(1):13-7.

76. Prabhakara Rao A. Oral, intramuscular and total dose infusion therapy of iron in iron deficiency anaemia. A comparative study. The Antiseptic. 1974;71(9):507-14.

77. Schwab J, Zitzmann H, Goltner E. Oral or intravenous iron therapy? (German). Med Welt (Die Medizinische Welt). 1973;24(19):797-9.

78. Myhre E. Duroferon--an iron preparation with delayed action [Norwegian] Duroferon--et jernpreparat med depotvirkning. Tidsskrift for den Norske laegeforening. 1973;93(35).

79. Mehta BC, Bhatt PD, Patel JC, Lotlikar K. Clinical trial of colloidal iron (saccharated oxide of iron) by oral route. Indian J Med Sci (Indian Journal of Medical Sciences). 1973;27(2):99-101.

80. Devarenne MF, Lemaire-Warembourg B, Marteau D, Delecour M. Trial of a new ferrous compound. Apropos of 17 cases of puerperal anemia (French) Essai d'un nouveau composé ferreux. A propos de 17 observations d'anémies des suites de couches. Lille Med (Journal de la Faculté de Médecine et de Pharmacie de l'Université de Lille). 1973;18(7):825-7.

81. Chamorro H, Moizeszowicz J. Treatment of hookworm anemia in a field study (author's transl). Bol Chil Parasitol (Boletín chileno de parasitología). 1973;28(1):24-30.

82. Chahud IA, Osorio NM. Iron deficiency anemia and its treatment with an iron sulfate preparation. Revista Clinica Espanola. 1973;131(1):61-4.

83. Krauss V, Nowotny D, Scharifzadeh AH. Effect and tolerance of oral iron therapy in the puerperium [German] Wirkung und Vertraglichkeit oraler Eisenbehandlung im Wochenbett. Munchener medizinische Wochenschrift (1950). 1972;114(43):1877-9.

84. Finzi C, Bailo U. Treatment of anemia in pregnancy: comparison of an iron delayed-action preparation and conventional preparations [Italian] Trattamento dell'anemia in gravidanza: confronto fra un preparato di ferro a cessione protratta ed i preparati convenzionali. Minerva Medica. 1972;63(43):2445-9.

85. Symonds EM, Radden HS, Cellier KM. Controlled-release iron therapy in pregnancy. The Australian & New Zealand journal of obstetrics & gynaecology. 1969;9(1):21-5.

86. Burslem Rw Fau - Poller L, Poller L Fau - Wacks H, H W. A trial of slow release ferrous sulphate (ferrogradumet) in prevention of iron deficiency in pregnancy. Acta Haematologica. 1968;40(4):200-4.

87. Reduced incidence of side-effects with an iron preparation. The Practitioner. 1967;198(188):845-8.

88. Crosland-Taylor P, Keeling DH, Cromie BW. A Trial of Slow-Release Tablets of Ferrous Sulphate. Curr Ther Res Clin Exp (Current Therapeutic Research, Clinical and Experimental). 1965;7:244-8.

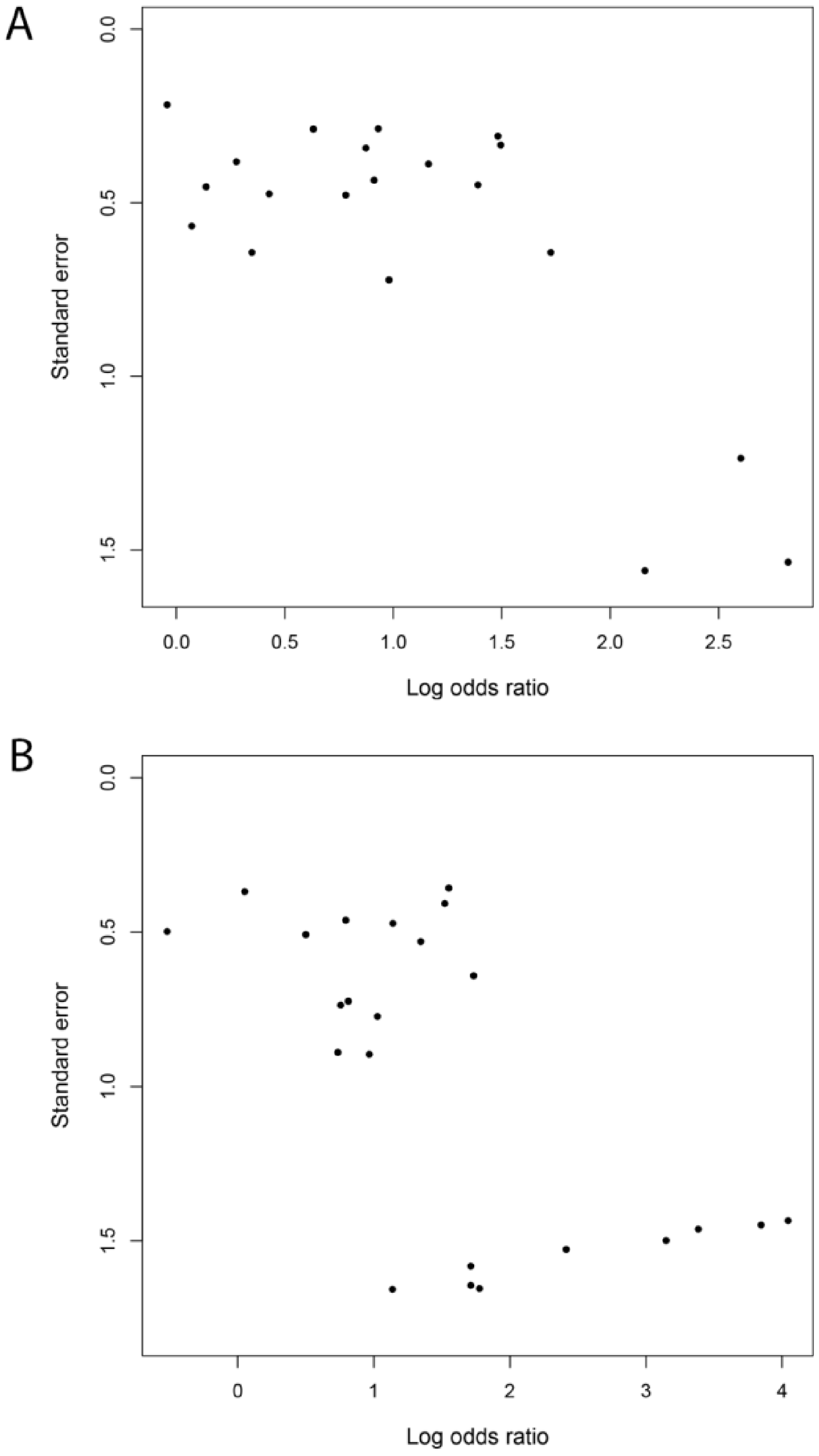

Figure A. Funnel plots of effect of daily ferrous sulfate supplementation on the incidence of gastrointestinal side-effects against standard error. (A) placebo-controlled trials (n=20) and **(B)** IV iron-controlled trials (n=23) were analysed separately. For each trial we plotted the effect as Log (OR) against its standard error.

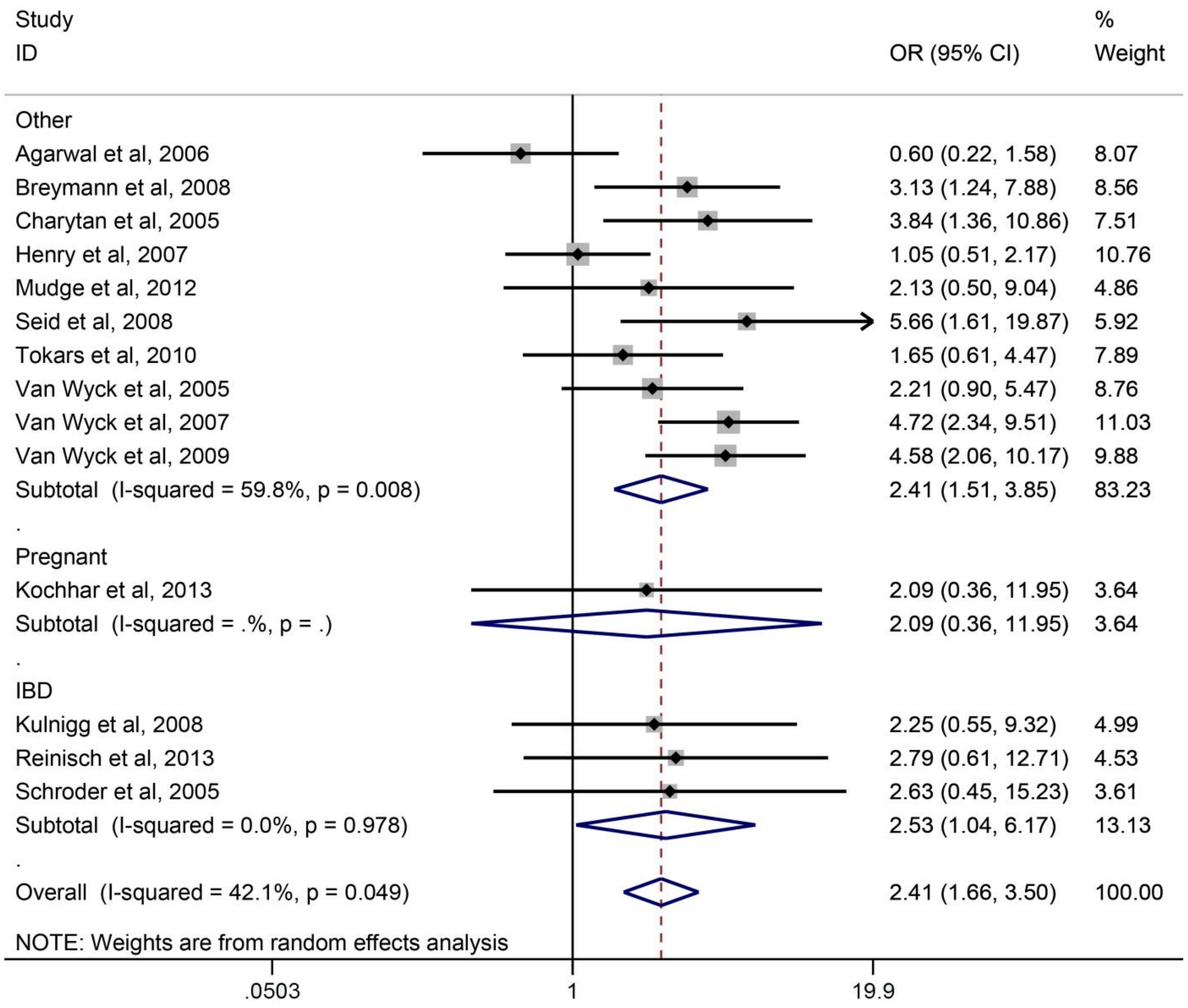

Figure B. Forest plot for the effect of daily ferrous sulfate supplementation on the incidence of gastrointestinal side-effects in IV iron-controlled RCTs. Only trials reporting one or more GI side-effect also for the IV iron comparator arm were included in this analysis, 9 studies were excluded in relation to Figure 3. Data for random-effects meta-analysis are shown. For each study the closed diamond represents the mean estimated effect and the horizontal lines the 95% CI. The grey shaded area surrounding each closed diamond represents the weight of each study in the analysis. Weight was assigned based on (inverse of) the sum of the within-study variance and between study variance. Open diamonds represent the subgroup mean difference and pooled overall mean differences as shown. Test for overall effect: z-score = 3.70 (other), 0.83 (pregnant), 2.04 (IBD), 4.64 (overall); *p*-value < 0.0001 (other), =0.4 (pregnant), =0.04 (IBD), <0.0001 (overall). IV, intravenous; OR, odds ratio; CI, confidence interval.

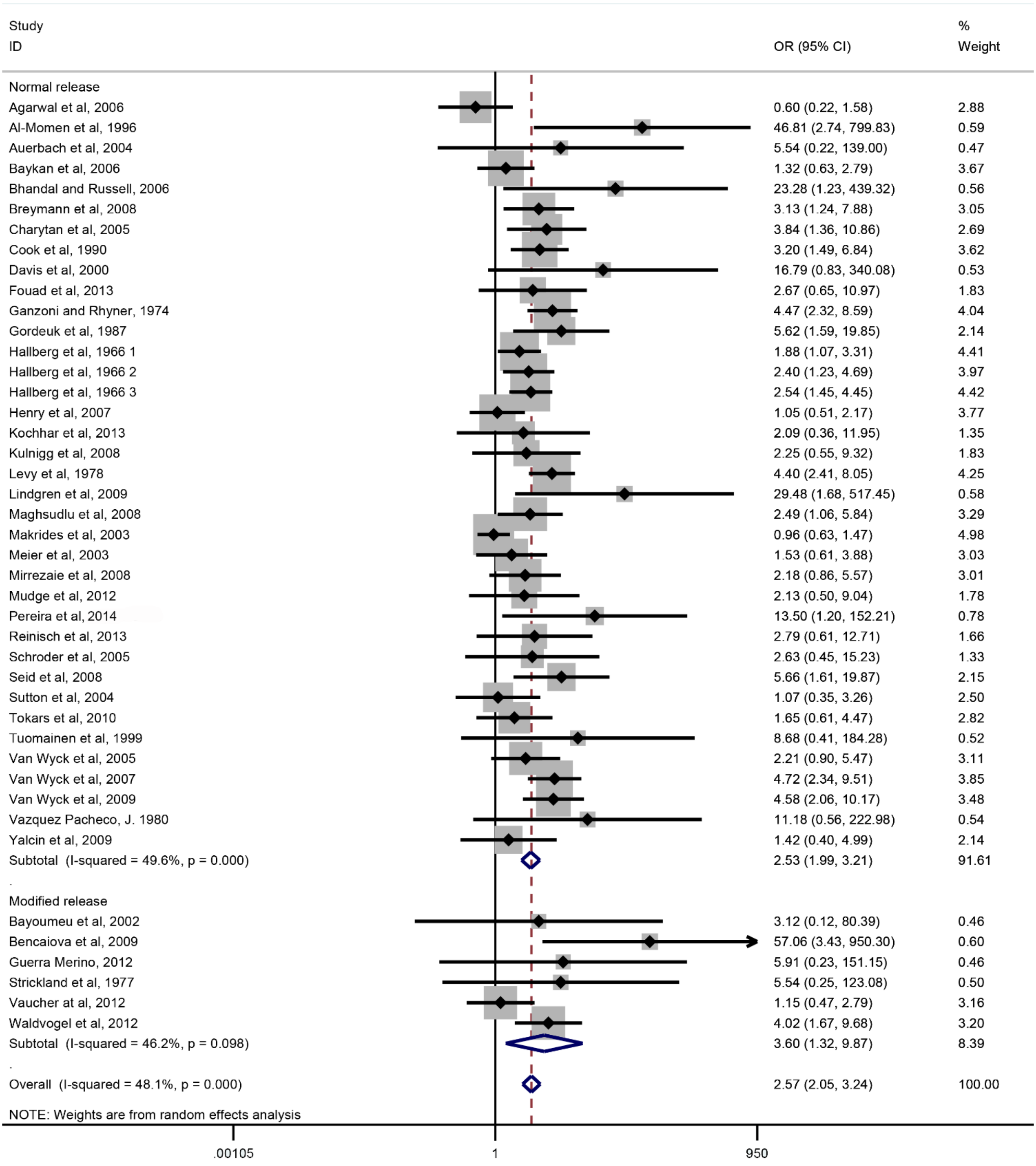

Figure C. Forest plot for the effect of daily ferrous sulfate supplementation on the incidence of gastrointestinal side-effects. Both placebo-controlled and IV iron-controlled trials were included in the analysis (43 RCTs, n=6831). Data for random-effects meta-analysis are shown. For each study the closed diamond represents the mean estimated effect and the horizontal lines the 95% CI. The grey shaded area surrounding each closed diamond represents the weight of each study in the analysis. Weight was assigned based on (inverse of) the sum of the within-study variance and between study variance. Open diamonds represent the subgroup mean difference and pooled overall mean differences as shown. Test for overall effect: z-score = 7.61 (normal-release), 2.49 (modified-release), 8.06 (overall); *p*-value <0.0001 (normal-release ferrous sulfate), =0.01 (modified-release ferrous sulfate), <0.0001 (overall). IV, intravenous; OR, odds ratio; CI, confidence interval.

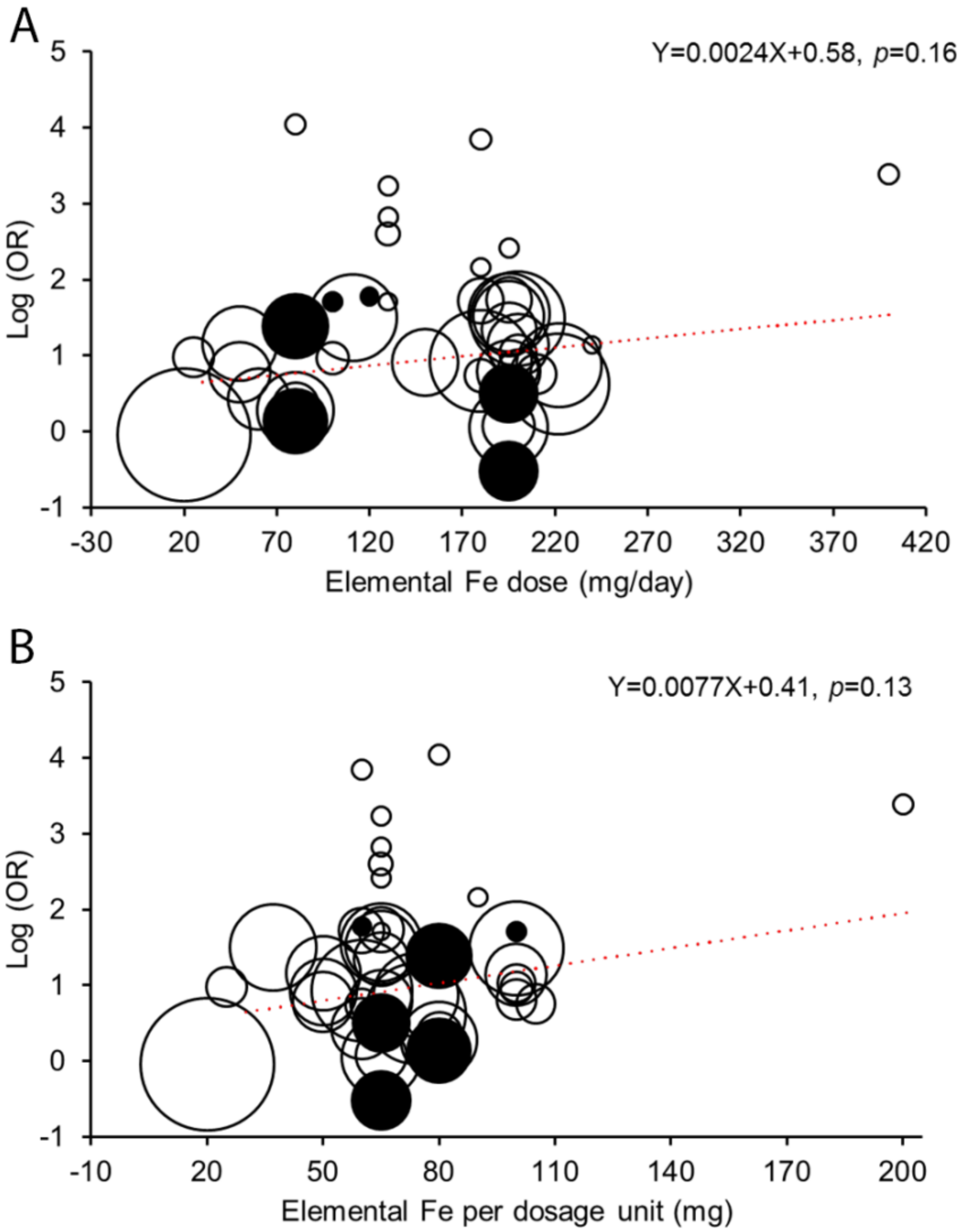

Figure D. Meta-regression analysis of the association between iron dosage and the odds ratio of gastrointestinal side-effects. Data from both placebo-controlled and IV iron-controlled trials were included in the analysis (43 studies, n=6831). **A,** meta-regression carried out for total daily elemental iron-equivalent dosage [*slope*=0.0024 (95% CI: -0.0009-0.0057); *t*-score=1.44, *p*=0.156]. **B,** meta-regression carried out for elemental iron-equivalent amount per dosage unit (typically per tablet or capsule) [*slope*=0.0077 (95% CI: -0.0022-0.0177); *t*-score=1.57, *p*=0.125].

Individual studies are represented by circles, with the size of the circle being inversely proportional to the variance of the estimated effect (i.e the larger the circle, the more precise the estimated effect). The dotted lines represent the regression line for the analysis. Closed circles, studies with modified release ferrous sulfate; open circles, studies with conventional ferrous sulfate (i.e. not modified-release). All studies used daily posology.
